# Integrated Bulk and Single-Cell Transcriptomic Analyses Identify a FOLR2^+^ Tissue-Resident Macrophage-Associated Lysophagy Gene Module in Heart Failure

**DOI:** 10.3390/genes17080957

**Published:** 2026-08-15

**Authors:** Qi Cheng, Yanli Wang, Deqiang Wang, Guoxing Wu, Biyun Liu, Qien Yuan, Fen Zhu

**Affiliations:** 1 School of Medicine, Wuhan University of Science and Technology, Wuhan 430081, China; chengqi19990117@wust.edu.cn (Q.C.); 15569292988@163.com (B.L.); yqe6667@163.com (Q.Y.); 2 School of Medicine, Jianghan University, Wuhan 430056, China; 13754865390@163.com (Y.W.); 17762527985@163.com (D.W.); wgx202513117@163.com (G.W.)

**Keywords:** heart failure, lysophagy, FOLR^2+^ tissue-resident macrophages, diagnostic biomarkers, single-cell RNA sequencing

## Abstract

**Objectives**: Heart failure (HF) arises from multiple interrelated pathological processes. Among these, lysosomal impairment and loss of autophagic homeostasis are increasingly recognized as important contributors to myocardial damage and ventricular remodeling. This study sought to identify lysophagy-associated signature genes in HF and to define their biological roles, cellular origins, and potential diagnostic relevance. **Methods**: Bulk myocardial transcriptome datasets, including GSE16499, GSE57338, and GSE76701, were integrated with the human cardiac single-cell dataset GSE145154. Differential expression analysis was first performed to identify lysophagy-related differentially expressed genes (DEGs). Candidate hub genes were then screened using support vector machine-recursive feature elimination (SVM-RFE) and least absolute shrinkage and selection operator (LASSO) regression. Functional enrichment analysis, Gene Set Enrichment Analysis (GSEA), immune infiltration assessment, single-cell transcriptomic mapping, and regulatory network analysis were subsequently conducted. The expression profiles of the selected genes were validated in a murine HF model, and VAMP8 overexpression assays were performed in H9c2 cells. **Results**: Five hub genes, namely *VAMP8*, *STX2*, *MCOLN1*, *DERL1*, and *PTP4A2*, were consistently and markedly decreased in failing myocardial tissue. These genes were mainly linked to SNARE-dependent vesicle trafficking and lysophagy regulation. A diagnostic model incorporating these hub genes demonstrated good discriminatory performance in both the training dataset and a small independent validation cohort, supporting further evaluation of their potential diagnostic value. Single-cell analysis further indicated that these genes were primarily enriched in cardiac FOLR2^+^ tissue-resident macrophages (TRMs). Pseudotime and cell–cell communication analyses associated this module with FOLR2^+^ TRM cell states and predicted interactions with cardiac stromal cells. In the HF mouse model, the mRNA levels of all five hub genes were decreased, with concurrent reductions in VAMP8, MCOLN1 and DERL1 protein expression. In Ang II/LLOMe-induced H9c2 cells, VAMP8 overexpression was associated with reduced cardiomyocyte injury, attenuation of changes in the abundance of lysosome- and autophagy-related proteins, and fewer ultrastructural abnormalities, suggesting a potential cardioprotective effect. **Conclusions**: *VAMP8*, *STX2*, *MCOLN1*, *DERL1*, and *PTP4A2* were identified as candidate molecular markers of HF that reflect alterations in a lysophagy- and vesicular-transport-related program associated with FOLR2^+^ tissue-resident macrophages. These findings provide new insights into immune-microenvironment remodeling in HF and suggest potential directions for mechanistic and therapeutic investigations.

## 1. Introduction

Heart failure (HF) is a heterogeneous clinical syndrome caused by reduced cardiac pumping capacity. During disease progression, compensatory mechanisms gradually become insufficient to maintain systemic metabolic requirements [1]. HF represents a substantial public health challenge worldwide and affects more than 64 million people [2]. As the global population continues to age, both the incidence of HF and HF-related mortality are expected to rise. At present, clinical diagnosis is mainly based on symptoms, physical signs, imaging evaluation, and biomarker measurements. B-type natriuretic peptide (BNP) and *N*-terminal pro-B-type natriuretic peptide (NT-proBNP) remain the most commonly applied biomarkers in clinical practice. Current treatment options include drug therapy, device-based intervention, and heart transplantation [3]. Nevertheless, existing diagnostic frameworks still have limitations in detecting early-stage disease and accurately predicting prognosis [4,5]. Further clarification of the molecular mechanisms involved in HF is therefore needed. The discovery of novel biomarkers with improved sensitivity and specificity may facilitate early screening, risk classification, and mechanism-guided therapeutic intervention.

The molecular pathogenesis of HF involves multiple pathological processes, including disordered energy metabolism, oxidative stress, endoplasmic reticulum stress, mitochondrial dysfunction, programmed cell death, and immune remodeling [6,7]. Abnormal autophagy has been shown to disrupt cardiomyocyte homeostasis and promote cardiac remodeling [8]. As the primary degradative organelles in cells, lysosomes require structural and functional integrity to maintain autophagic flux, protein quality control, and cellular metabolic homeostasis. Lysophagy is a form of selective autophagy that eliminates damaged lysosomes and maintains lysosomal quality control [9]. This selectivity distinguishes lysophagy from macroautophagy, which delivers a broader range of cytoplasmic cargo to lysosomes. Following lysosomal membrane damage, exposed luminal glycans are recognized by cytosolic galectins. Limited damage may be repaired by the ESCRT machinery, whereas irreparable lysosomes become ubiquitinated and are subsequently removed by lysophagy [9,10,11]. VCP/p97 appears to act downstream of autophagy-receptor recruitment and contributes to efficient lysophagic flux [9]. TFEB-mediated activation of the CLEAR network and lysosomal biogenesis then helps restore the lysosomal pool [12]. If damaged lysosomes are not efficiently repaired or removed, continued leakage of lysosomal contents can disrupt mitochondrial function and sustain cellular stress and inflammatory signaling [13]. Accumulating evidence suggests that lysosomal membrane injury, together with defective lysosomal repair and clearance, can promote cardiomyocyte damage and impair cardiac function [12,14]. However, in contrast to canonical autophagy pathways, the expression characteristics, diagnostic value, and cellular distribution of lysophagy-related genes (LRGs) in HF have not been fully defined.

In this study, integrated bioinformatics analyses were applied to identify lysophagy-related hub genes associated with HF. A diagnostic model was then established to assess the biomarker potential of these candidate genes. Functional annotation, immune infiltration analysis, single-cell localization, and regulatory network construction were further used to explore their biological significance and cell-type specificity. Finally, the expression patterns of the selected genes were experimentally validated in a mouse model of HF. In addition, VAMP8 overexpression was examined in H9c2 cells to determine whether it could alleviate cardiomyocyte injury and attenuate lysosomal dysfunction. Overall, this study identified candidate biomarkers and molecular features that may help clarify HF pathogenesis and provide potential directions for early intervention and targeted therapeutic strategies.

**Figure 1:**
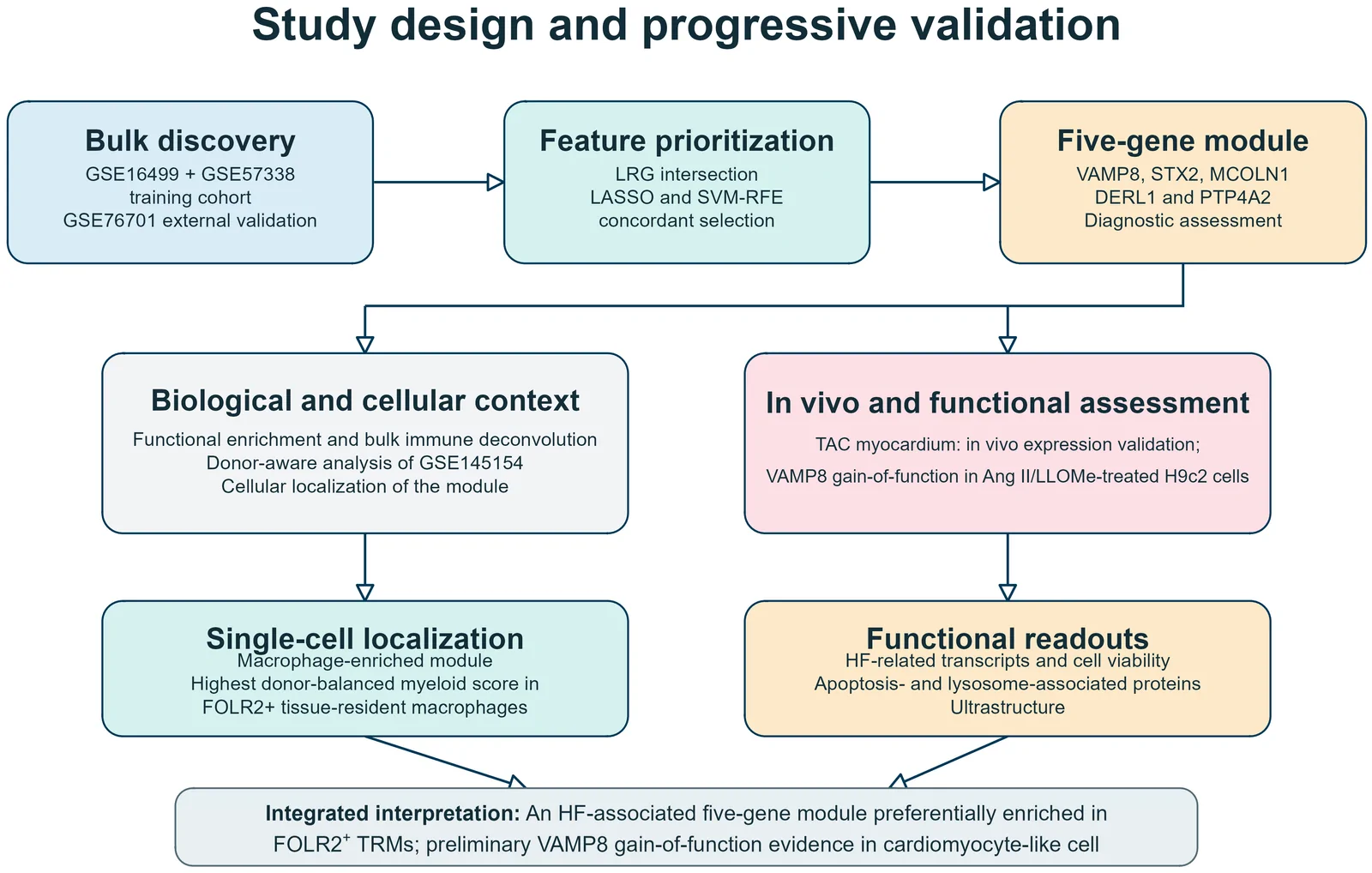
Study design and evidence integration framework. Bulk transcriptomic discovery followed by machine-learning prioritization yielded a five-gene module. Single-cell analysis characterized its cellular distribution, TAC myocardium validated the in vivo expression pattern, and VAMP8 overexpression in Ang II/LLOMe-injured H9c2 cells provided preliminary gain-of-function evidence.

A workflow diagram of the study is shown in Figure 1.

## 2. Materials and Methods

### 2.1. Data Acquisition

Public transcriptomic datasets were obtained from the Gene Expression Omnibus (GEO) database [15]. Three microarray datasets of myocardial tissue were included: GSE16499, GSE57338, and GSE76701. GSE16499 contained 15 HF and 15 control samples, GSE57338 contained 177 HF and 136 control samples, and GSE76701 contained 4 HF and 4 control samples. GSE16499 and GSE57338 were combined as the training cohort, whereas GSE76701 was used as an external validation cohort. The single-cell dataset GSE145154, which included myocardial scRNA-seq data from 5 patients with HF and 1 control subject, was used for single-cell analysis (Table 1).

LRGs were collected from GeneCards using the keyword “lysophagy,” and only protein-coding genes with a relevance score > 1 were retained. Additional LRGs reported in previous studies were identified through PubMed using the same keyword [16]. After merging the two sources and removing duplicates, 105 LRGs were included for subsequent analysis (Supplementary Table S1).

For the bulk transcriptomic analysis, the merged training cohort was normalized and corrected for batch effects using the sva package [17] and limma (version 3.58.1) [18]. Data quality and batch-correction performance were evaluated using expression distribution plots and principal component analysis (PCA).

### 2.2. Identification of LRDEGs

Using the limma software (version 3.58.1) [18], we were able to identify DEGs between the HF and control samples in the merged dataset. Adjusted *p* < 0.05 and |log2FC| > 0.25 were used as the significance thresholds [19]. With the help of ggplot2 (version 3.4.4), volcano plots were created. LRDEGs were defined as the intersection of DEGs and LRGs. Pheatmap (version 1.0.12) was used to produce heatmaps, and RCircos (version 1.2.2) was used to display chromosomal localization [20].

### 2.3. Validation and Analysis of LRDEG Expression

Group comparison plots were used to compare the expression levels of the LRDEGs between the HF and control groups in the combined dataset. To measure the degree of association between each pair of LRDEGs, we ran a Spearman correlation analysis and used igraph (version 1.6.0) and ggraph (version 2.1.0) to create correlation heatmaps. Gene Ontology (GO) and Kyoto Encyclopedia of Genes and Genomes (KEGG) analyses were performed using clusterProfiler (version 4.10.0) [21] to investigate molecular functions and processes, with adjusted *p* < 0.05 and FDR q value < 0.25 as thresholds.

### 2.4. GSEA

ClusterProfiler (version 4.10.0) [21] was used to conduct GSEA with all genes in the combined dataset ranked by logFC. We only allowed sets of genes with 10–500 genes. We used the Molecular Signatures Database (MSigDB; Broad Institute, Cambridge, MA, USA) c2.all.v2024.1.Hs.symbols.gmt collection to get our background gene sets. After applying the Benjamini–Hochberg (BH) adjustment, a significance level was considered to be adjusted *p* < 0.05 and a q value < 0.25.

### 2.5. Identification of Candidate Biomarkers and Construction of the Diagnostic Model

To choose candidate feature genes, two machine learning (ML) algorithms, LASSO regression and SVM-RFE, were employed following the identification of LRDEGs. Implementing LASSO regression with set.seed (2000) and family = “binomial” was done using the R package glmnet (version 4.1-8) [22]. We used tenfold cross-validation to get the best lambda and kept the genes whose coefficients were not zero. In order to build SVM classification models utilizing recursive feature elimination, the e1071 software was used to execute SVM-RFE. Finding the sweet spot between accuracy and error rate in tenfold cross-validation led to the ideal number of genes to use. Genes identified by both methods were referred to as candidate hub genes. The intersection, rather than the union, was used to prioritize features consistently selected by both algorithms and reduce method-specific selection bias. Area under the curve (AUC) was computed using the pROC program (version 1.18.5) [23], and ROC curves were used to evaluate the diagnostic performance of specific genes.

### 2.6. Model Validation and External Validation

The five hub genes were used as independent variables in a diagnostic prediction model for HF, with sample categorization serving as the dependent variable. Using the rms package (version 6.7-1), a nomogram was created to estimate the risk of HF. Decision curve analysis (DCA), calibration curves, and receiver operating characteristic (ROC) curves were used to evaluate the model’s performance. Independent validation on the GSE76701 dataset followed evaluation in the combined training cohort.

### 2.7. Single-Gene GSEA

We studied each hub gene independently to explore its potential association with HF-related biological processes. The expression level of each gene was considered a continuous variable, and the hub gene and all the genes in the training set were correlated using Spearman’s coefficients. Following this, the genes were sorted based on the association coefficients. We used clusterProfiler (version 4.10.0) [21] to conduct single-gene GSEA, following the same analytical parameters outlined in Section 2.4.

### 2.8. Immune Infiltration Analysis

We used the CIBERSORT algorithm and the LM22 reference matrix to determine the relative proportions of 22 immune cell types. The analysis focused on comparing the immune cell composition of HF and control samples. Further evaluation was conducted using Spearman correlation analysis to assess associations between hub gene expression and immune cell fractions. We used ggplot2 (version 3.4.4) and pheatmap (version 1.0.12) to create heatmaps and other related visualizations.

### 2.9. Single-Cell RNA Sequencing Analysis

To identify the cellular sources of the hub genes, the human myocardial single-cell RNA sequencing dataset GSE145154 was reanalyzed. This dataset contained 24 FACS-sorted libraries from 6 human myocardial samples, including 12 CD45+ immune cell libraries and 12 CD45- nonimmune cell libraries. Each library was imported as a raw 10× Genomics count matrix. Cells were excluded if they met any of the following quality-control criteria: hemoglobin gene proportion > 5%, ribosomal gene proportion > 40%, mitochondrial gene proportion ≥ 10%, UMI count < 1000, or detected gene count < 500. Doublets were detected and removed separately for each library using scDblFinder. After quality control, 81,757 high-quality cells were retained for downstream analysis.

The CD45+ and CD45- fractions were normalized, log1p-transformed, and subjected to highly variable gene selection. PCA was then performed, and donor-associated batch effects were corrected using Harmony, with donor ID specified as the batch variable. Unsupervised clustering was conducted with the Leiden algorithm. No formal parameter-optimization procedure was performed. The resulting clusters were evaluated and annotated based on cluster-specific genes and canonical cell-type markers. Cell populations were visualized by UMAP and annotated using automated CellTypist annotation, COSG-derived cluster-specific marker genes, and manual curation based on canonical cell-type markers. The activity of the five-gene hub module was quantified using the rank-based AUCell algorithm. Cell types were ranked according to donor-balanced mean module activity. Overall differences were assessed using Friedman repeated-measures tests, followed by paired Wilcoxon signed-rank tests with Benjamini–Hochberg correction for pairwise comparisons. Because the dataset included only one control donor, disease-associated differential analyses were interpreted descriptively.

Myeloid cells from the CD45+ fraction were further extracted and reclustered using a Leiden resolution of 0.8. The resulting subclusters were annotated according to cluster-specific genes and canonical myeloid markers. Myeloid subpopulations, including FOLR2^+^/LYVE1+ TRMs, were annotated according to subcluster marker genes. Pseudotime trajectories were inferred using Slingshot and Palantir, with classical monocytes defined as the starting population. These trajectory results were interpreted descriptively. Ligand–receptor communication analysis was performed using LIANA, with emphasis on the microenvironmental interaction network involving FOLR2^+^ TRMs. All single-cell analyses were conducted in Python using OmicVerse v2.2.0, Scanpy v1.11.5, harmonypy v2.0.0, scDblFinder (pyscdblfinder v0.1.0), CellTypist v1.7.1, COSG v1.0.4, LIANA v1.7.3, Palantir v1.4.4, and Slingshot (pyslingshot v0.2.0).

### 2.10. Regulatory Network Construction

Transcription factors (TFs) regulate gene expression by binding to promoters or enhancers. This work used ChIPBase, TarBase, and the Comparative Toxicogenomics Database (CTD) to predict TFs, miRNAs, and medicines or chemicals that could be linked to the probable hub genes, respectively. Predictions for TFs were considered significant at *p* < 0.05, while predictions for drugs or compounds were considered significant at *p* < 0.01. Cytoscape (version 3.10.2) was used to build and display the mRNA-TF, mRNA-miRNA, and mRNA-drug interaction networks [24].

### 2.11. Transverse Aortic Constriction (TAC)-Induced Mouse Model of HF

Beijing Jinglai Huake Biotechnology Co., Ltd. (Beijing, China) supplied twelve male C57BL/6J mice that were specific-pathogen-free. The mice were 6–8 weeks old and weighed 20–22 g. All exploratory procedures were carried out in compliance with relevant animal ethical regulations, and the study protocol was approved by the Animal Ethical Committee of Beijing Jinglai Huake Biotechnology Co., Ltd. (approval No. JLHK-20251209-01). Six mice each were placed in a sham-operated group and six mice into a TAC group. Aortic constriction caused HF in the TAC group. The sham group of mice had the identical operation but did not have their aortas constricted.

Echocardiography was performed using a Vevo 2100 ultrasound imaging system (FUJIFILM VisualSonics, Toronto, ON, Canada) to assess heart function eight weeks following surgery, and body weight (BW) was documented. The measured parameters included left ventricular end-systolic diameters (LVEDs), end-diastolic diameters (LVEDd), fractional shortening (FS), ejection fraction (EF), left ventricular end-diastolic volume (LVEDV), left ventricular end-systolic volume (LVESV), and left ventricular mass (LVW). Mice were euthanized after echocardiographic evaluation, and their hearts were collected for Western blotting (WB) and RT-qPCR.

### 2.12. VAMP8 Overexpression in H9c2 Cells

Originating in Shanghai, China, the H9c2 cells were obtained from the Cell Bank/Stem Cell Bank of the Chinese Academy of Sciences. H9c2 cells were cultured in DMEM (Procell, Wuhan, China) supplemented with 10% fetal bovine serum (FBS) and 1% penicillin–streptomycin at 37 °C in a humidified atmosphere containing 5% CO_2_. Using Lipofectamine 3000 (Invitrogen Carlsbad, CA, USA), pcDNA3.1-VAMP8 plasmids were transfected into cells to overexpress VAMP8 (OE). The cells that were used as a negative control (Vector) were those that had pcDNA3.1-NC plasmids transfected.

Following transfection, Vector- and OE-transfected cells were treated with angiotensin II (Ang II, 1 μM) for 48 h, with 0.75 mM LLOMe added during the final hour to induce lysosomal damage, and were designated as the Model and OE + Model groups, respectively. Corresponding Vector and OE control groups received vehicle. The CCK-8 assay was used to evaluate cell viability. The data were adjusted to the Vector group after detecting absorbance at 450 nm.

### 2.13. RT-qPCR Analysis

In order to determine the levels of candidate genes’ mRNA expression in the cardiac tissues of Sham and TAC mice, RT-qPCR was used. Additionally, it was utilized to evaluate the *Vamp8*, *NPPA*, *NPPB*, and *MYH7* expression levels in H9c2 cells. A NanoPhotometer N50 (Implen GmbH, Munich, Germany) was used for the quantification of total RNA, which was extracted using TRIzol reagent from Solarbio in Beijing, China. cDNA was synthesized from messenger RNA by employing a reverse transcription kit manufactured by Yeasen in Wuhan, China. Next, the expression levels of *PTP4A2*, *MCOLN1*, *DERL1*, *VAMP8*, *STX2*, *NPPA*, *NPPB*, and *MYH7* were detected by RT-qPCR.

*GAPDH* was used as the internal reference gene. Relative gene expression was calculated using the 2^−ΔΔCt^ method. Statistical analysis and visualization were performed using ggplot2 (version 3.4.4), stats (version 4.2.1), and car (version 3.1-0). Primer sequences are provided in Table 2.

### 2.14. WB

The following proteins were measured in cardiac tissues using WB: DERL1, MCOLN1, and VAMP8; in H9c2 cells, VAMP8, cleaved caspase-3, LAMP2, CTSB, ACP2, LAMP1, LC3B, p62, and TFEB. Total protein concentration was measured using a protein assay kit (Fubio, Beijing, China). Protein samples were separated by 10% SDS-PAGE and transferred onto nitrocellulose membranes. Primary antibodies against DERL1 (HUABIO, Hangzhou, China), MCOLN1 and VAMP8 (Proteintech, Wuhan, China), cleaved caspase-3, LAMP2, CTSB, ACP2, LAMP1, LC3B, p62, and TFEB (Abmart, Shanghai, China) were incubated overnight at 4 °C on membranes that had been blocked with 5% nonfat milk for 2 h. After that, the appropriate secondary antibodies were added to the membranes for incubation. Protein bands were visualized using a a Tanon 5200 chemiluminescence imaging system (Tanon, Shanghai, China). The band intensities were measured using ImageJ (version 1.53k; National Institutes of Health, Bethesda, MD, USA), and β-actin or GAPDH was utilized as the internal control.

### 2.15. Transmission Electron Microscopy (TEM)

Cardiomyocytes underwent ultrastructural examination using TEM. Centrifugation was used to create cell pellets from each group’s carefully collected cells. The pellets were postfixed in 1% osmium tetroxide after being promptly fixed in 2.5% glutaraldehyde at 4 °C. Ultrathin slices were cut from the samples after they were dehydrated using a graded ethanol series. The samples were then embedded in epoxy resin. Prior to imaging, the sections were stained with lead citrate and uranyl acetate and then studied using an HT7800 transmission electron microscope (Hitachi High-Tech, Tokyo, Japan). TEM was performed in the Vector, OE, Model, and OE + Model groups, and the images were used for qualitative comparison of basal and injury-associated ultrastructural changes.

### 2.16. Statistical Analysis

With the exception of single-cell workflows, all data preprocessing and statistical procedures were performed in R software (version 4.2.2). For two-group comparisons, variables satisfying normality assumptions were assessed with Student’s *t* test, whereas variables with non-normal distributions were examined using the Mann–Whitney U test. Differences across three or more groups were analyzed with the one-way ANOVA followed by Tukey’s post hoc test or Kruskal–Wallis test. Associations between variables were determined by Spearman’s rank correlation analysis. Multiple-testing adjustment was performed using the Benjamini–Hochberg procedure. Except for TEM, all in vitro quantitative experiments were independently repeated three times using separate biological replicates. TEM was used as a representative ultrastructural observation and was not included in quantitative statistical analysis. Statistical significance was defined as *p* < 0.05. * *p* < 0.05; ** *p* < 0.01; *** *p* < 0.001.

## 3. Results

### 3.1. Integration and Normalization of Myocardial Expression Profiles

**Figure 2:**
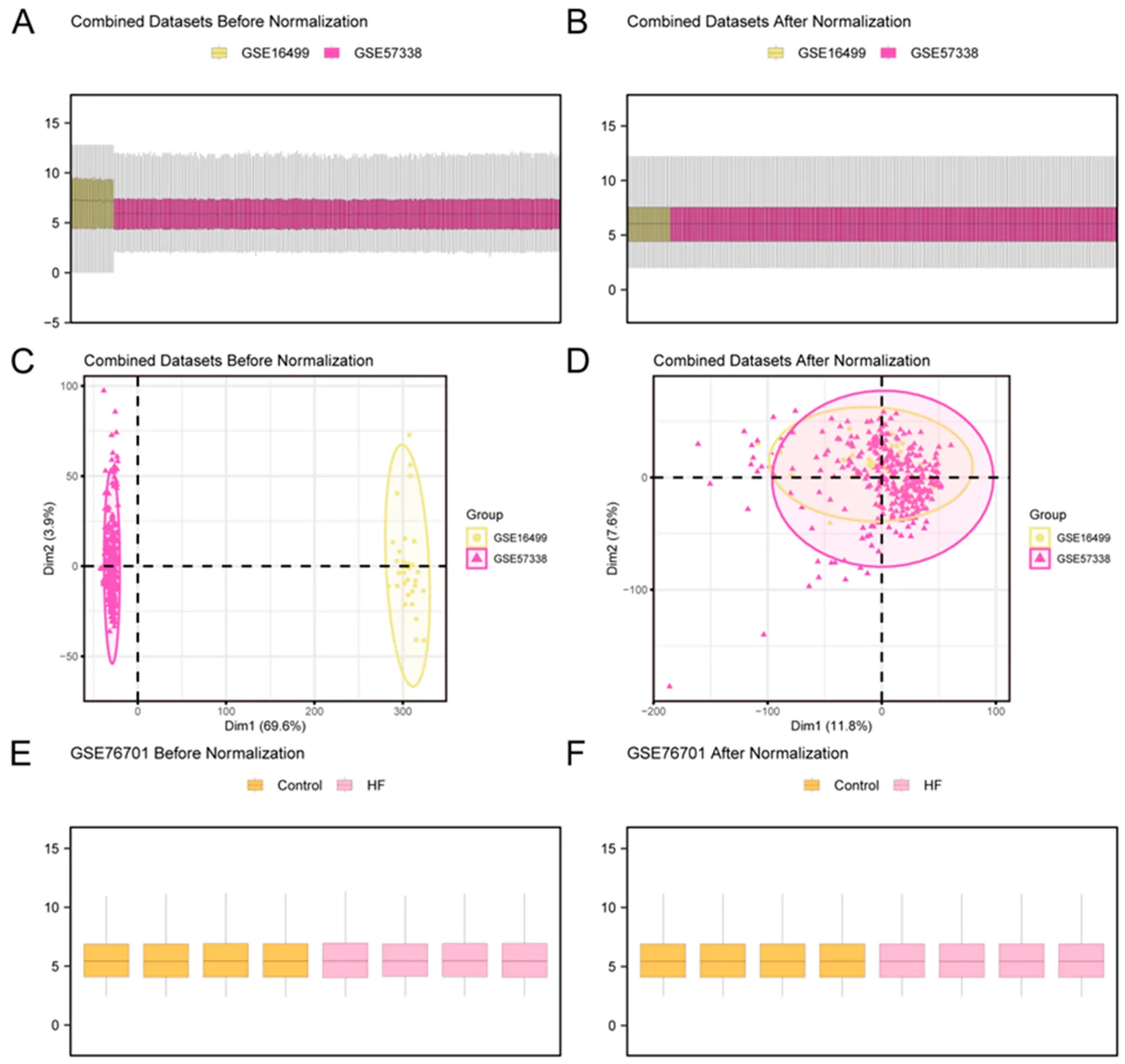
Normalization and batch-effect adjustment of HF-associated expression datasets. (**A**,**B**) Boxplots depicting the expression profiles of the merged GSE16499 and GSE57338 dataset before and after normalization. (**C**,**D**) PCA plots of the integrated dataset before and after correction for batch effects. (**E**,**F**) Boxplots showing expression distributions before and after normalization of the external validation dataset GSE76701. In the combined dataset, pink denotes GSE57338 and light yellow denotes GSE16499; in GSE76701, yellow denotes control samples and pink denotes HF samples. In panels (**A**,**B**,**E**,**F**), the gray whiskers indicate the ranges of the sample-wise expression distributions.

To improve the stability of differential expression analysis, GSE16499 and GSE57338 were normalized and corrected for batch effects to generate the training set. Boxplots showed that expression distributions became more consistent after correction (Figure 2A,B). PCA further showed that sample separation attributable to dataset origin was substantially reduced after batch correction (Figure 2C,D). In the external validation dataset GSE76701, overall expression distributions were comparable between the HF and control groups after normalization and correction (Figure 2E,F). These findings indicated that the integrated dataset was of sufficient quality for subsequent differential expression analysis.

### 3.2. Identification and Expression Characteristics of LRDEGs

**Figure 3:**
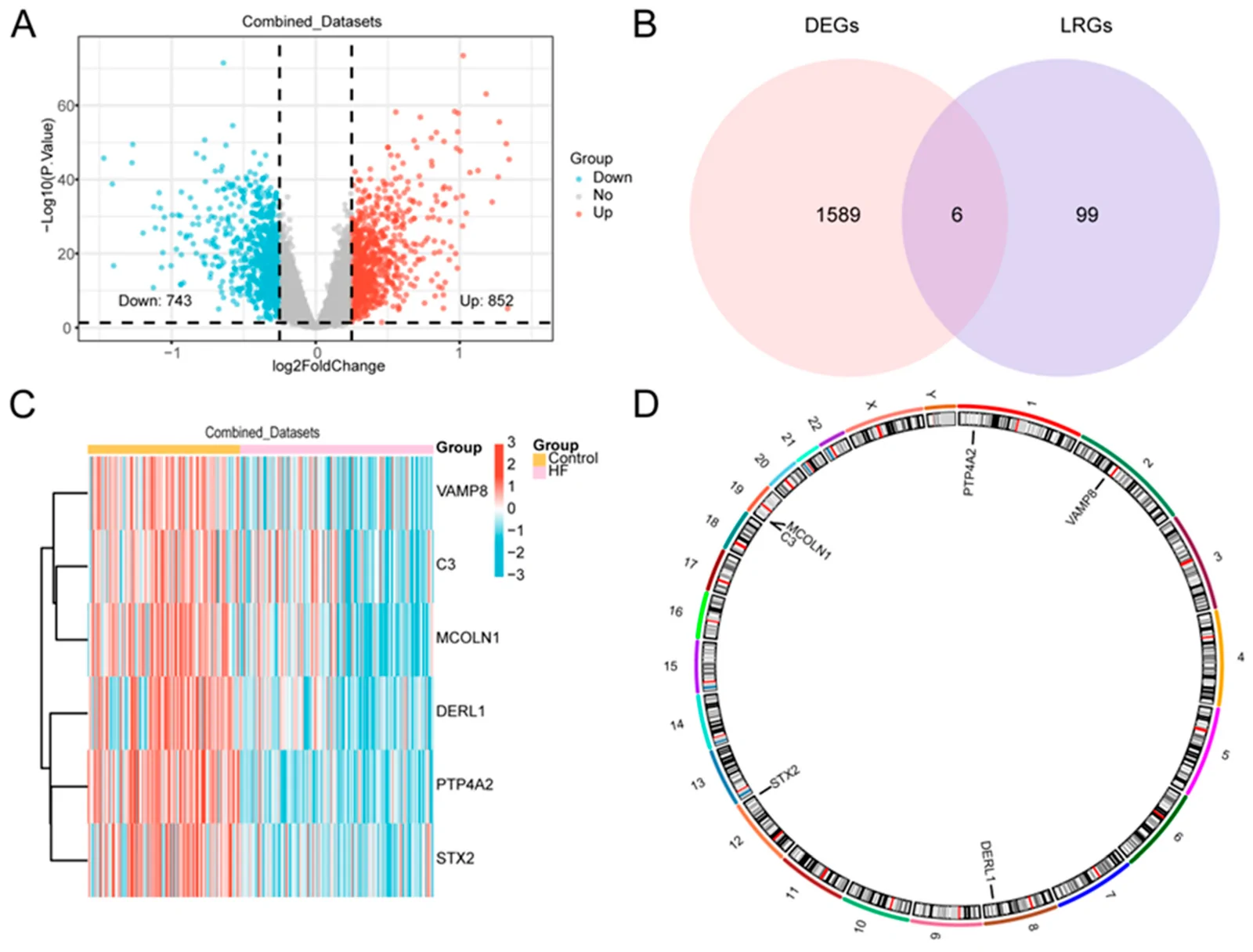
Screening and characterization of LRDEGs. (**A**) Volcano plot of DEGs between HF and control samples. (**B**) Venn diagram showing the intersection of DEGs and LRGs. (**C**) Heatmap showing LRDEG expression in the combined dataset. (**D**) Chromosomal localization of LRDEGs. In panel (**A**), the black dashed lines indicate the log2 fold-change and adjusted *p*-value thresholds.

Differential expression analysis of the training set identified 1595 DEGs (Figure 3A). Intersecting these DEGs with the 105 LRGs yielded 6 LRDEGs: *VAMP8*, *C3*, *MCOLN1*, *DERL1*, *PTP4A2*, and *STX2* (Figure 3B). Heatmap analysis showed distinct expression patterns for these 6 genes between the control and HF groups (Figure 3C). Chromosomal localization analysis showed that the 6 LRDEGs were distributed across different chromosomes (Figure 3D), indicating that they were not derived from a single chromosomal cluster.

**Figure 4:**
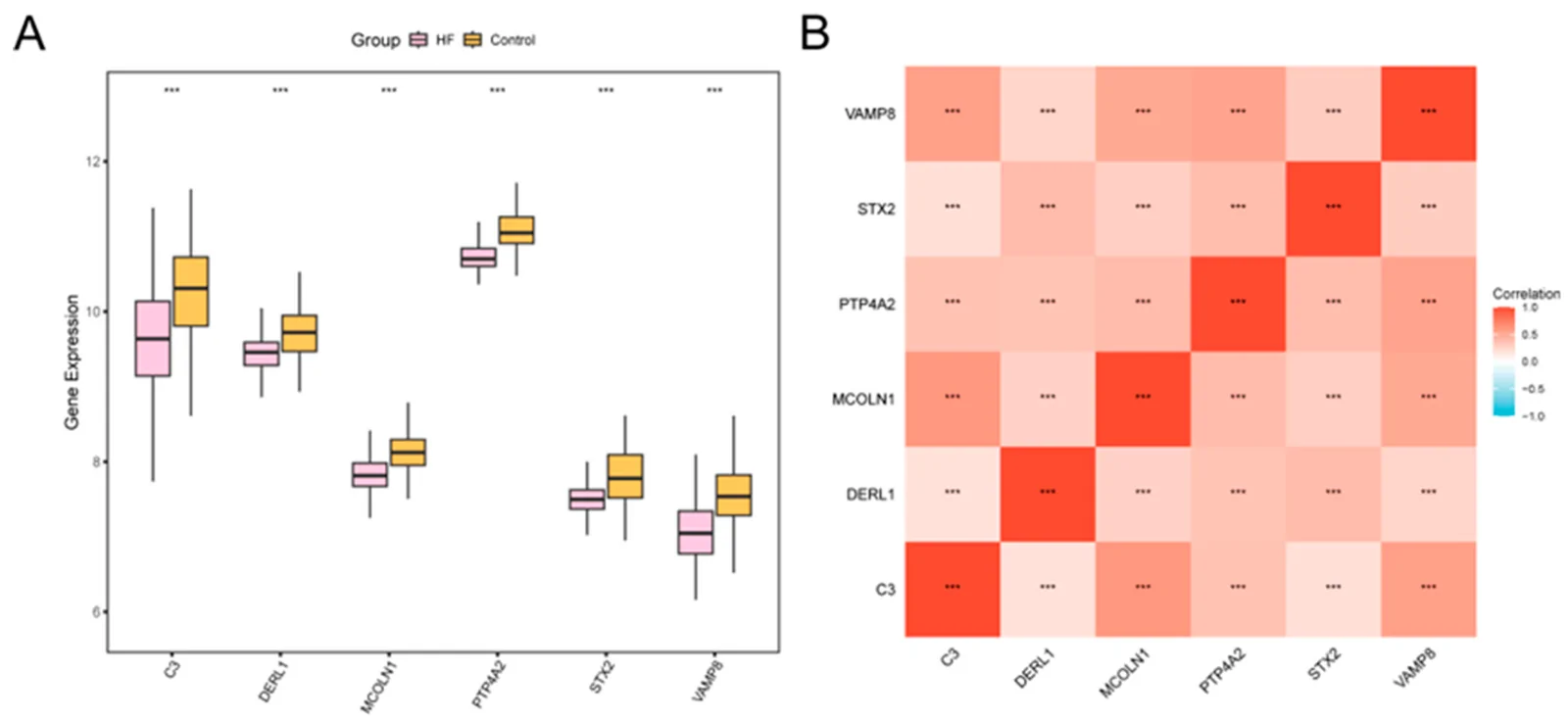
Validation of LRDEG expression differences and correlation analysis. (**A**) Expression levels of the 6 LRDEGs in HF and control groups in the combined dataset. (**B**) Correlation heatmap of the 6 LRDEGs. *** *p* < 0.001.

Further comparison showed that all 6 LRDEGs were expressed at lower levels in HF samples than in control samples (Figure 4A). Spearman correlation analysis showed predominantly positive correlations among the 6 LRDEGs (Figure 4B), indicating that these genes may constitute a coordinately regulated lysophagy-related module in failing myocardium.

### 3.3. Functional Enrichment Analysis of Candidate LRDEGs

**Figure 5:**
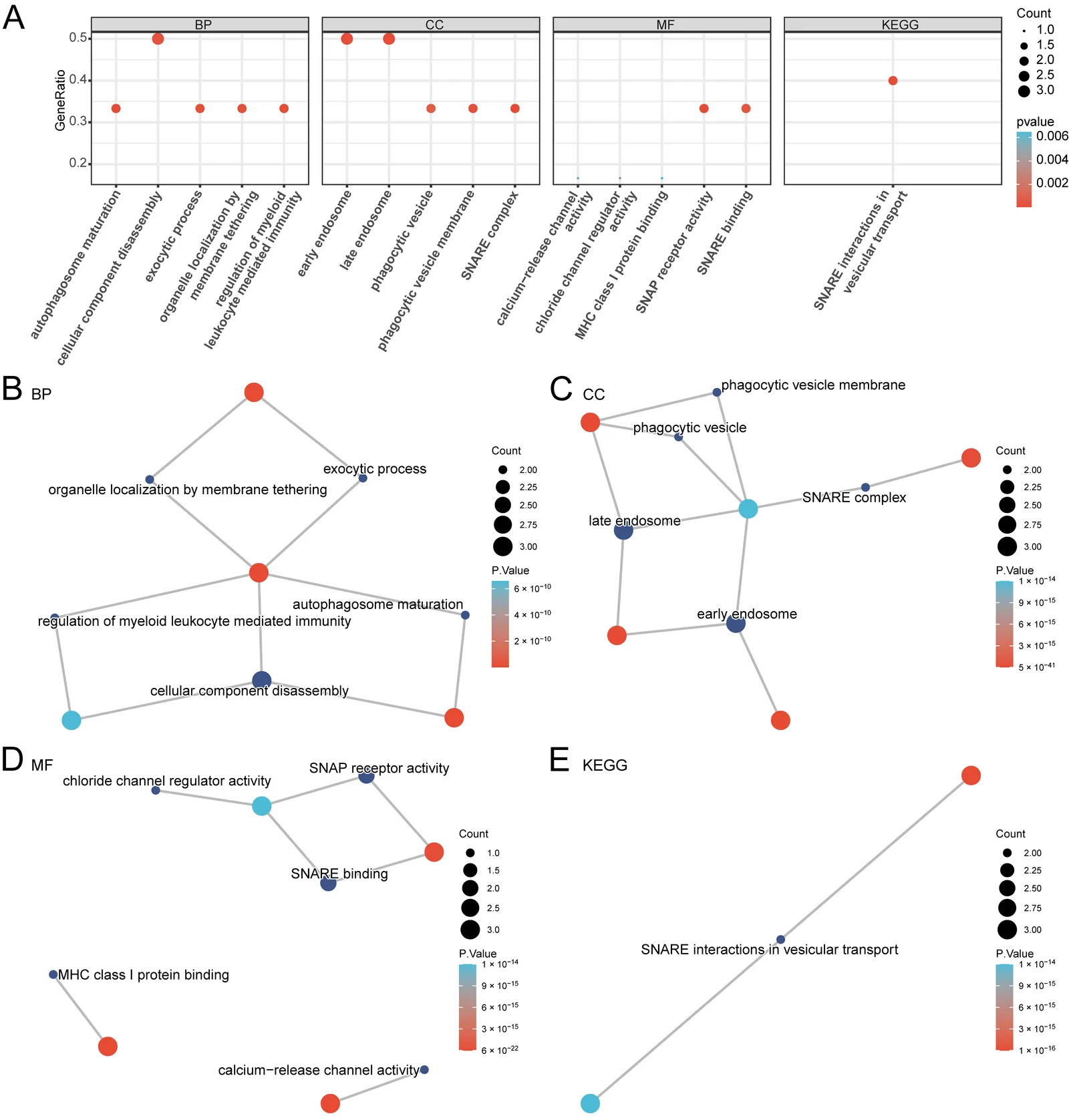
GO and KEGG analyses of LRDEGs. (**A**) Bubble plot of GO and KEGG results; the *x*-axis indicates enriched functional terms. (**B**–**E**) Network plots showing enrichment in BP, CC, MF, and KEGG categories. Bubble or node size indicates gene count, and color encodes the *p* value.

GO and KEGG analyses were conducted to characterize the biological functions of candidate LRDEGs (Supplementary Table S2). GO terms were mainly enriched in autophagosome maturation, cellular component disassembly, exocytosis, immune regulation, and membrane-organelle localization at the biological process (BP) level; early endosomes, late endosomes, phagocytic vesicle membranes, and SNARE complexes at the cellular component (CC) level; and SNAP receptor activity, SNARE binding, chloride channel regulator activity, and calcium-release channel activity at the molecular function level (Figure 5A–D). KEGG analysis further identified significant enrichment in SNARE interactions in vesicular transport (Figure 5E). Overall, these results indicate disruption of coordinated lysosomal and vesicular trafficking rather than an isolated defect in canonical autophagy.

### 3.4. ML-Based Identification of HF Hub Genes and Construction of the Diagnostic Model

**Figure 6:**
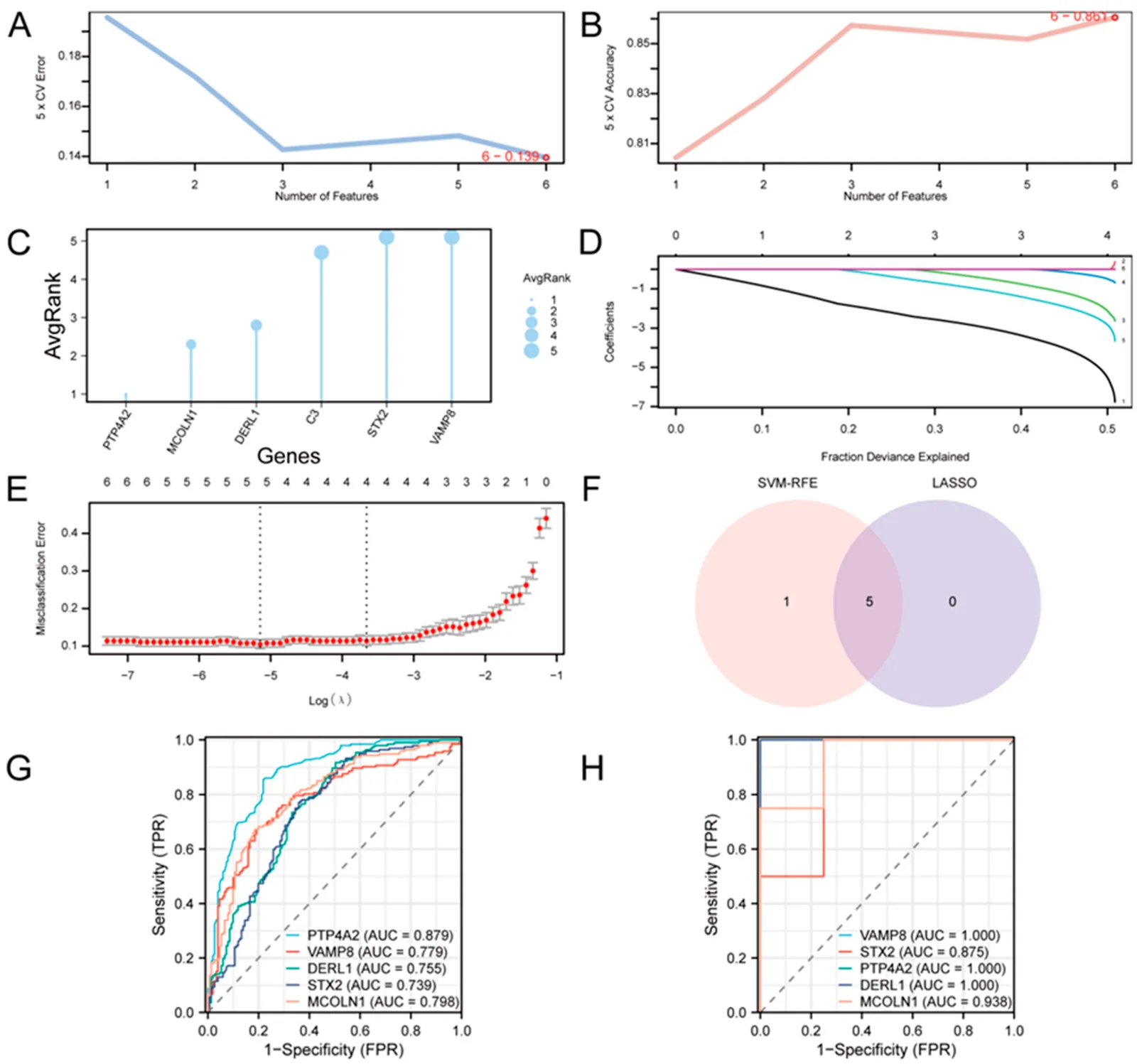
ML-based feature selection and diagnostic evaluation of LRDEGs. (**A**–**C**) SVM-RFE algorithm: (**A**) cross-validation (CV) error curve, (**B**) CV accuracy curve, and (**C**) candidate gene ranking by average rank (AvgRank). (**D**,**E**) LASSO regression: (**D**) coefficient trajectory plot and (**E**) cross-validation error curve. (**F**) Venn diagram showing the overlap between genes selected by SVM-RFE and LASSO regression. (**G**,**H**) ROC curves of candidate hub genes in the training set (**G**) and validation set (**H**). In panel (**B**), the red point marks the selected feature set at maximum cross-validation accuracy. In panels (**G**,**H**), the gray dashed diagonal denotes the no-discrimination reference, and the colored curves correspond to the genes shown in the legend.

Based on the LRDEGs identified above, SVM-RFE and LASSO regression were used to further select HF-related hub genes from the LRDEGs. SVM-RFE identified 6 candidate feature genes: *PTP4A2*, *MCOLN1*, *DERL1*, *C3*, *STX2*, and *VAMP8* (Figure 6A–C). LASSO regression, using the minimum lambda criterion as the optimal solution, selected 5 feature genes: *PTP4A2*, *MCOLN1*, *DERL1*, *STX2*, and *VAMP8* (Figure 6D,E). The intersection of these two approaches yielded 5 HF-related hub genes: *PTP4A2*, *MCOLN1*, *DERL1*, *VAMP8*, and *STX2* (Figure 6F). These genes were subsequently considered candidate diagnostic biomarkers. ROC analysis showed that all five genes had discriminatory value in both the training and validation sets, with single-gene AUC values exceeding 0.7 (Figure 6G,H). These five genes were therefore carried forward for subsequent pathway, immune infiltration, single-cell, and experimental analyses.

**Figure 7:**
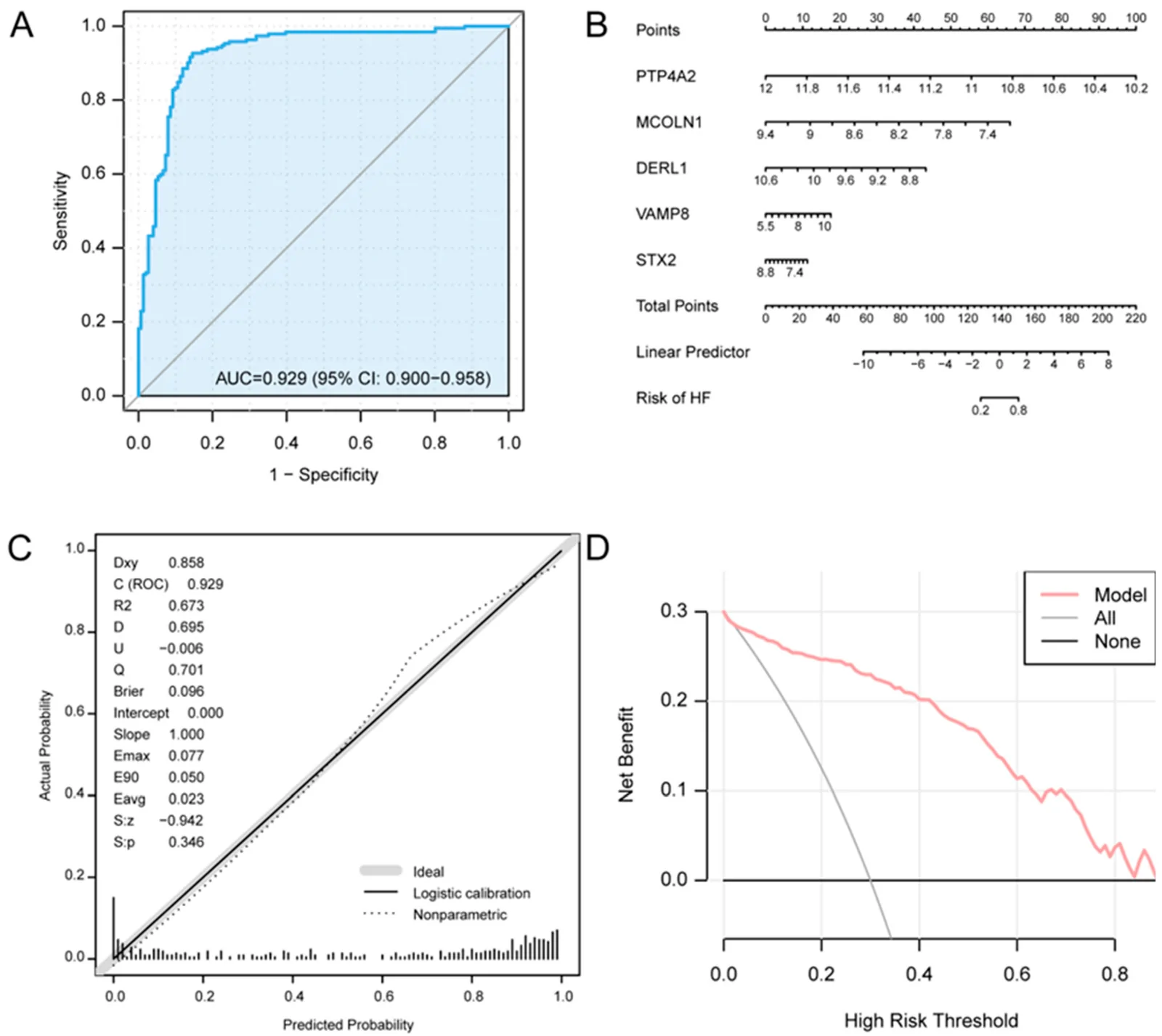
Development and assessment of the HF diagnostic model in the training cohort. (**A**) ROC curve for the five-gene diagnostic classifier in the training cohort. (**B**) Nomogram constructed from the five hub genes. (**C**) Calibration plot of the diagnostic model. (**D**) DCA of the model.

A diagnostic classifier was subsequently developed based on the five hub genes. In the training cohort, this model yielded an AUC greater than 0.9, indicating high apparent discrimination (Figure 7A). The nomogram visualized the relative weight of each gene in the prediction model, among which *PTP4A2* contributed the most and *STX2* contributed the least (Figure 7B). Calibration analysis showed agreement between the predicted risks and the observed outcomes in the training cohort (Figure 7C). Decision curve analysis further showed that the model produced a higher net benefit than both the “treat-all” and “treat-none” approaches over a wide interval of threshold probabilities, supporting further evaluation in appropriately sized independent clinical cohorts (Figure 7D).

**Figure 8:**
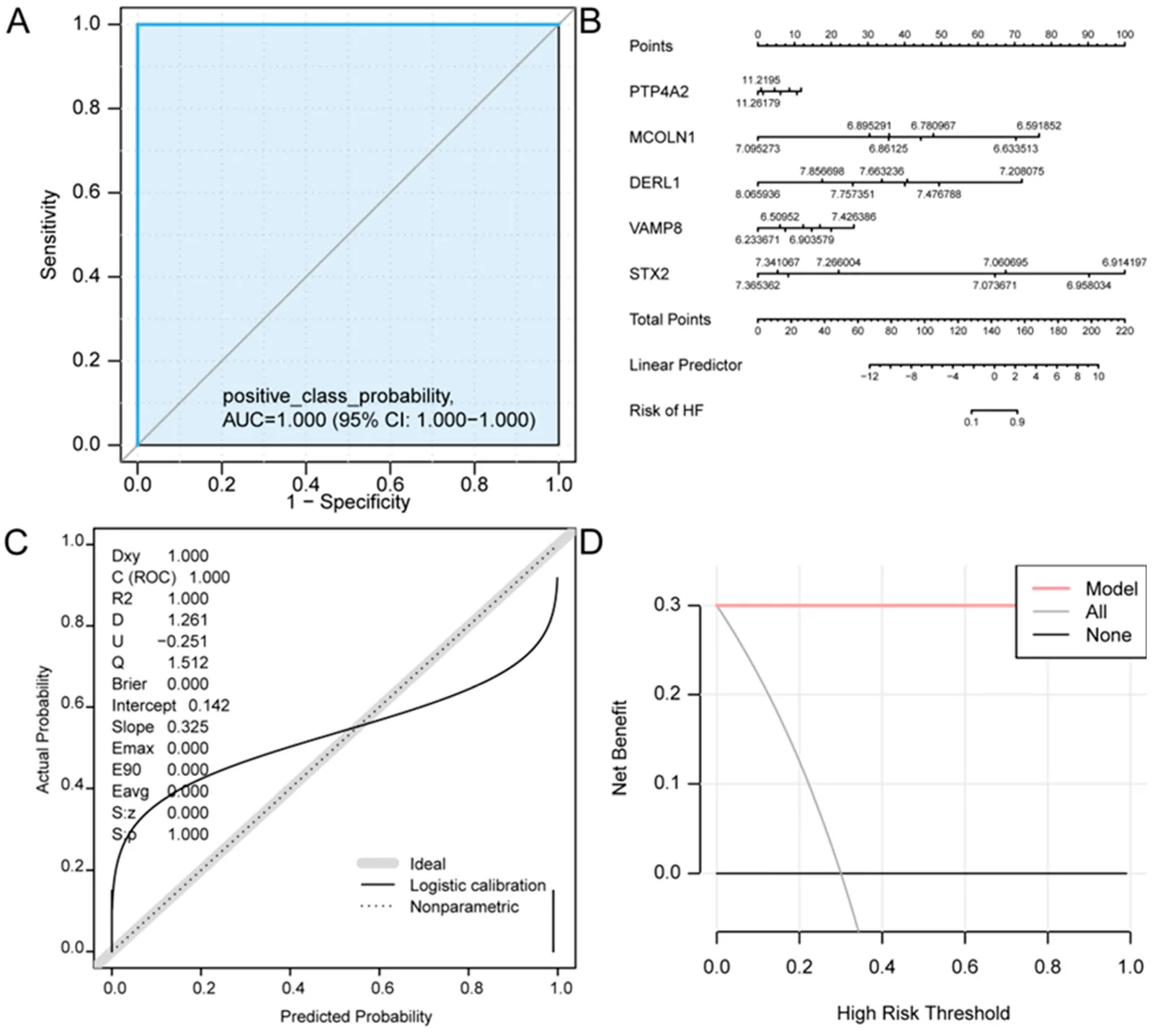
Validation of the HF diagnostic model in the external GSE76701 dataset. (**A**) ROC curve in the external validation set. (**B**) Nomogram based on the five hub genes. (**C**) Calibration curve. (**D**) DCA.

To assess whether the model retained predictive performance beyond the training cohort, external validation was performed. In the eight-sample GSE76701 cohort, the model achieved an AUC > 0.9. Calibration and decision-curve plots were also generated (Figure 8). However, because of the extremely small sample size, these analyses should be considered exploratory and cannot establish model stability or clinical generalizability.

### 3.5. HF-Related Pathways and Immune Infiltration Characteristics

**Figure 9:**
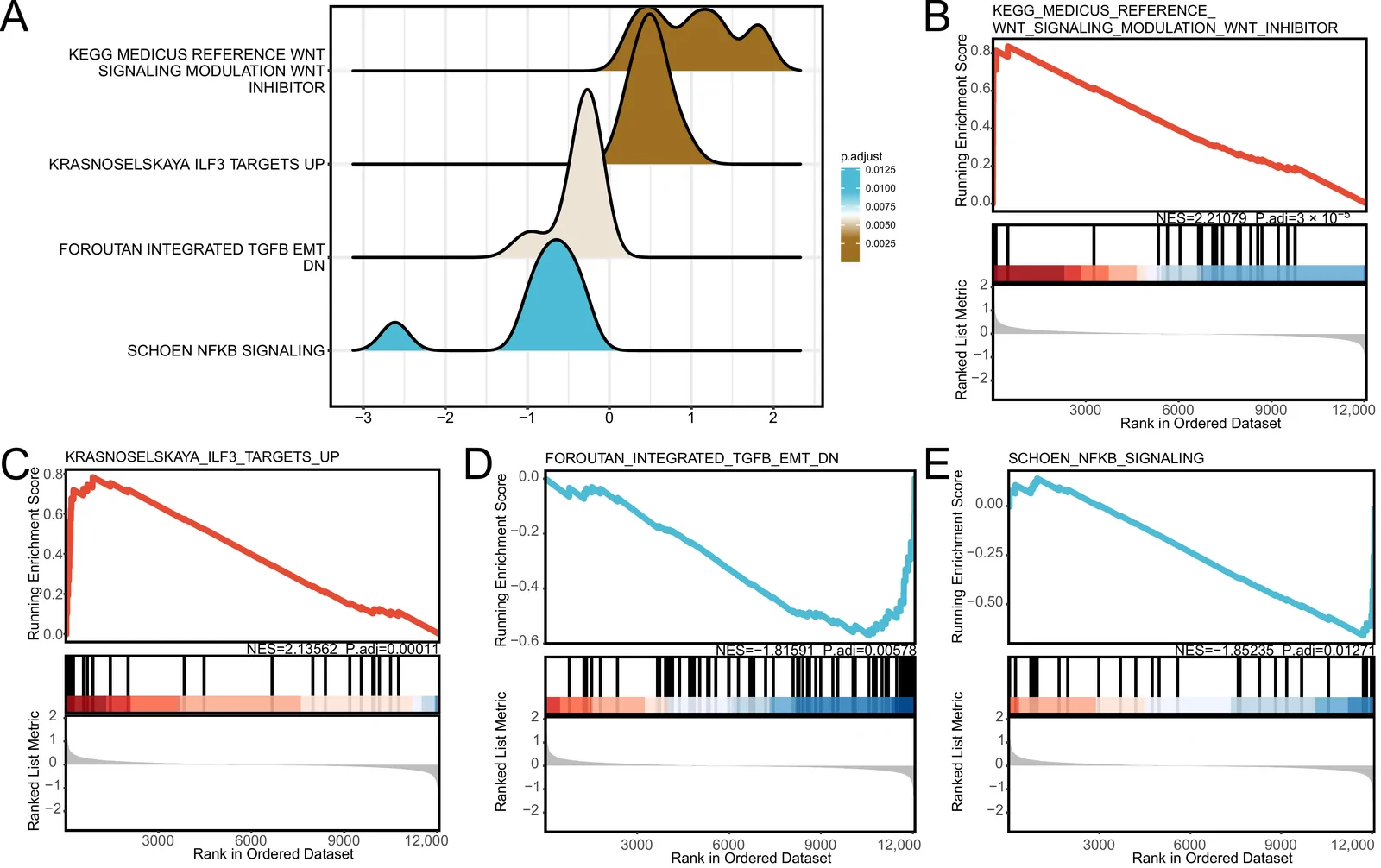
GSEA of the integrated dataset. (**A**) Ridge plot showing significantly enriched gene sets in the combined dataset. (**B**–**E**) GSEA enrichment curves for representative gene sets. In panel (**A**), ridge fill color represents the adjusted *p* value. In panels (**B**–**E**), red enrichment-score curves indicate positive enrichment and blue curves indicate negative enrichment; the red-to-blue ranked-list color strip represents the direction and magnitude of the ranked metric, black vertical bars mark gene-set hits, and the gray area shows the ranked-list metric distribution.

GSEA was performed using the integrated training set to identify pathways associated with HF (Figure 9A, Supplementary Table S3). Genes in HF samples were significantly enriched in gene sets related to regulation of WNT signaling, ILF3 targets, TGF-β/EMT-associated processes, and NF-κB signaling (Figure 9B–E). These results suggest that HF progression is accompanied by dysregulation of pathways involved in myocardial fibrosis, extracellular matrix remodeling, chronic inflammation, and immune regulation. Several enriched gene sets were originally derived from cancer-related studies. However, the underlying transcriptional programs are shared across multiple diseases and are not specific to cancer. In the present study, these gene sets were therefore interpreted in the context of inflammation, extracellular matrix remodeling, and cellular stress.

Single-gene GSEA was then performed to characterize the potential pathways associated with each hub gene. The results indicated that the candidate genes were linked to distinct biological processes. *VAMP8*, *DERL1*, *MCOLN1*, and *PTP4A2* were mainly associated with inflammatory and immune responses, matrix remodeling, and stress-response pathways, whereas *STX2* was linked to mitochondrial respiration and energy metabolism (Supplementary Figure S1). Reduced expression of these five hub genes may reflect impaired autophagy-lysosomal homeostasis, immune homeostasis, and mitochondrial metabolism in failing myocardium.

**Figure 10:**
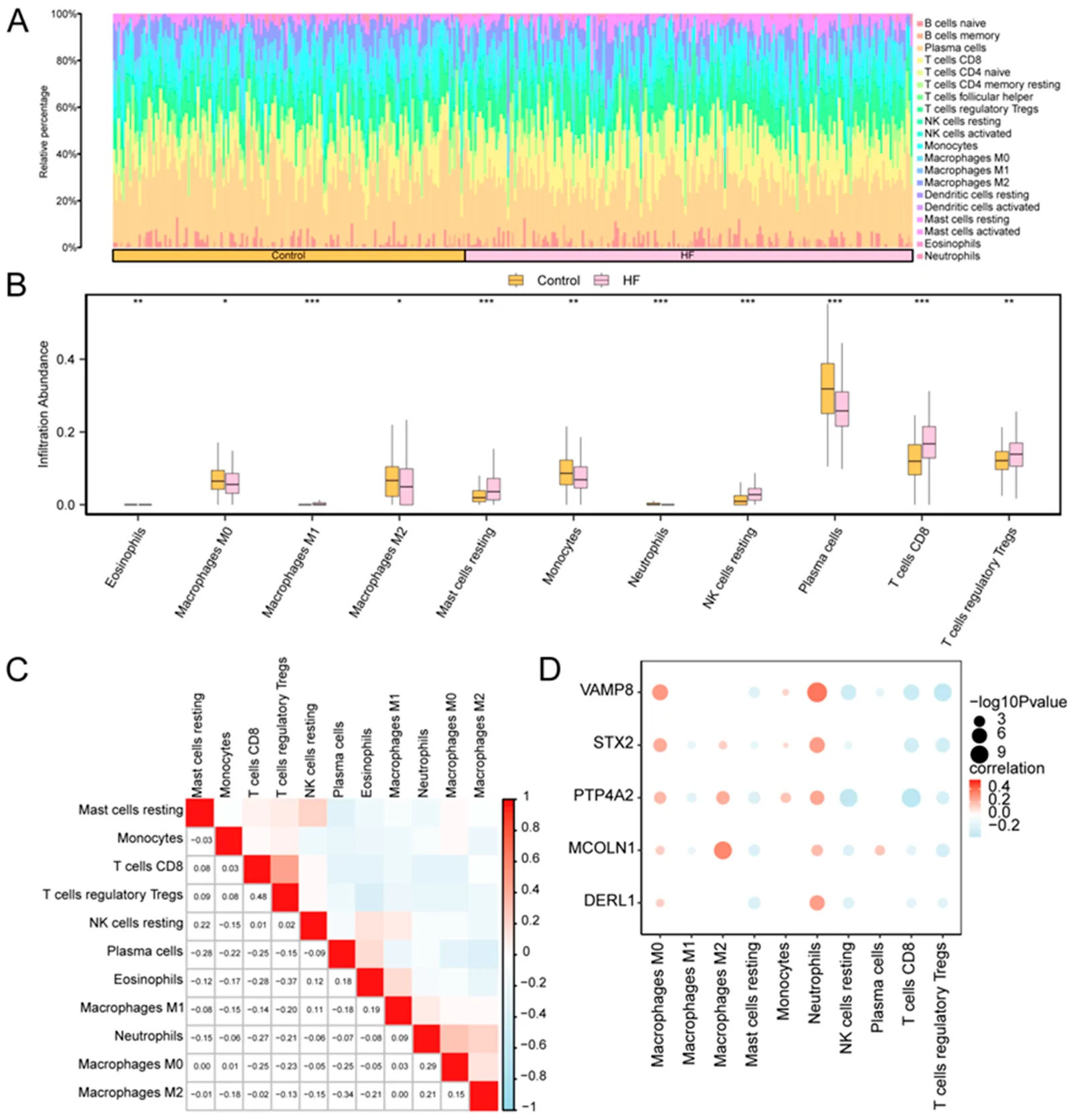
Immune infiltration patterns and their associations with hub genes in HF. (**A**) Relative proportions of 22 immune cell types in HF and control samples. (**B**) Immune cell subsets with significant intergroup differences. (**C**) Correlation heatmap showing relationships among immune cell fractions. (**D**) Bubble plot depicting associations between hub gene expression and immune cell subsets. * *p* < 0.05; ** *p* < 0.01; *** *p* < 0.001.

Immune processes play an important role in the onset and progression of HF. Accordingly, CIBERSORT was applied to infer the relative proportions of 22 immune cell populations in HF and control samples (Figure 10A). Among these immune components, 11 cell subsets showed significant differences between the two groups (Figure 10B). Correlation analysis further revealed heterogeneous relationships among the estimated immune cell fractions (Figure 10C). For the hub genes, *VAMP8* displayed the strongest positive association with M2 macrophages (*r* = 0.54, *p* < 0.05), whereas *PTP4A2* exhibited the most pronounced inverse association with activated CD8+ T cells (*r* = −0.35, *p* < 0.05) (Figure 10D). These results suggest that the candidate genes may participate in the remodeling of the myocardial IME in HF. However, these bulk-level associations could not identify the cell populations in which the five-gene signature was concentrated.

### 3.6. Single-Cell Analysis Reveals Enrichment of Hub Genes in Cardiac Macrophages and FOLR2^+^ TRM Subpopulations

**Figure 11:**
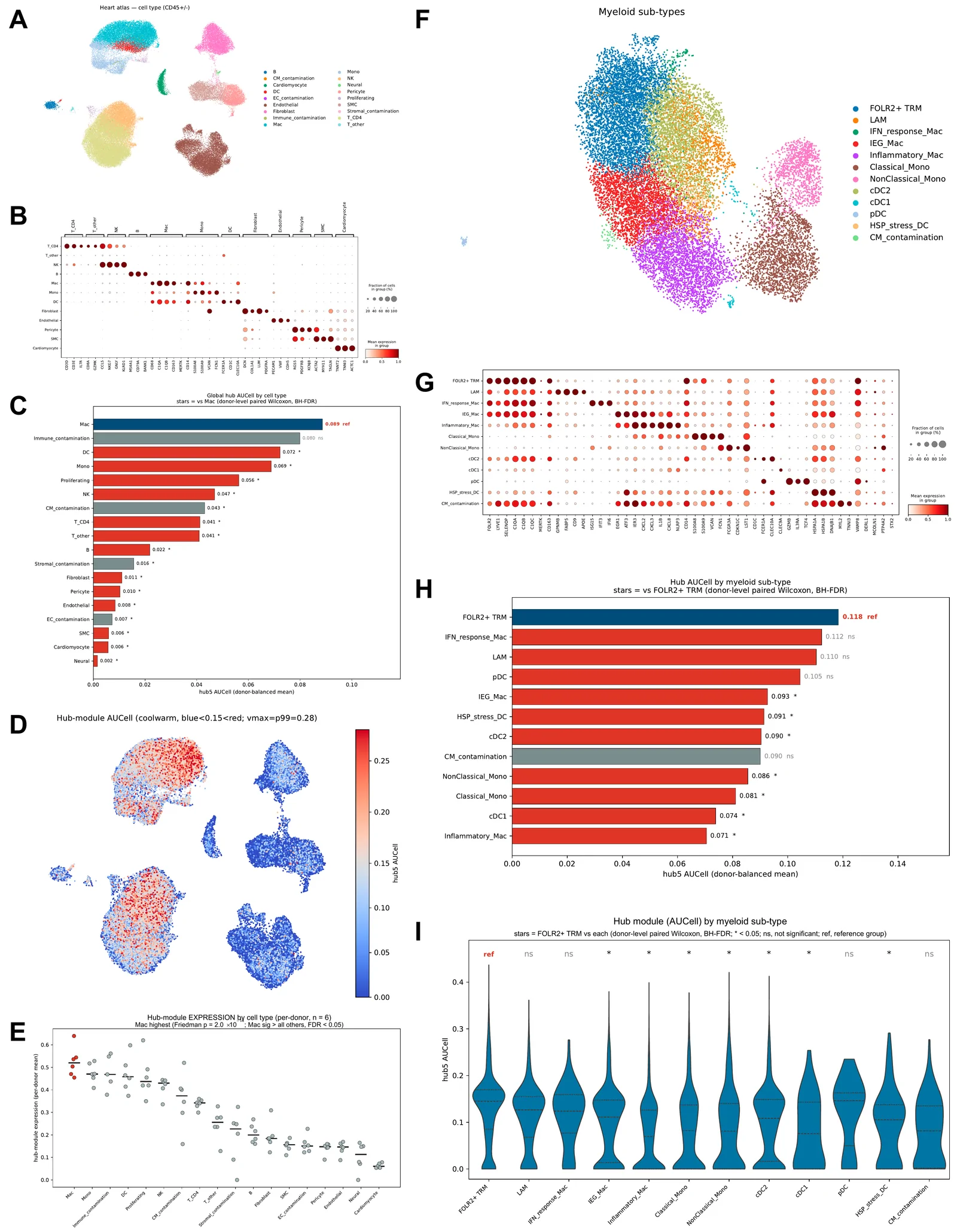
Cell-type localization of candidate hub genes in the cardiac single-cell atlas. (**A**) UMAP plot of the combined CD45+/CD45- cardiac single-cell atlas with cell-type annotation. (**B**) Dot plot showing canonical marker gene expression across cell types. (**C**) Donor-balanced mean ranking of AUCell activity for the hub gene module by cell type. (**D**) UMAP visualization of AUCell activity for the hub gene module using a coolwarm color scheme and a threshold of 0.15. (**E**) Donor-level expression of the hub gene module. (**F**) UMAP plot of cardiac myeloid cell subclusters. (**G**) Dot plot showing myeloid subtype markers and hub gene expression; *VAMP8* showed the highest expression in FOLR2^+^ TRMs. (**H**) Donor-balanced ranking of AUCell activity by myeloid subtype. (**I**) Violin plots showing AUCell activity across myeloid subtypes. In panels (**B**,**G**), dot size indicates the fraction of cells expressing a gene and dot color indicates mean expression. In panels (**C**,**H**), blue denotes the reference group, red denotes a significant difference, and gray denotes a nonsignificant difference. * *p* < 0.05; ns, not significant; ref, reference group.

To resolve the cellular distribution of the bulk-derived five-gene hub signature, the human myocardial scRNA-seq dataset GSE145154 was reanalyzed. After integration of CD45+ and CD45- data, quality control, doublet removal, normalization, batch correction, and clustering, a cardiac single-cell atlas containing 81,757 cells was constructed. UMAP visualization showed clearly separated cell populations, and major cardiac cell types were annotated according to canonical marker expression (Figure 11A,B). To reduce the influence of dropout events, the AUCell algorithm was used to calculate module activity for the five hub genes, namely *VAMP8*, *STX2*, *MCOLN1*, *DERL1*, and *PTP4A2*. This module showed the highest activity in macrophages (Figure 11C). Individual gene UMAP and dot-plot analyses further showed that *VAMP8* had the strongest macrophage-enriched expression, whereas the other four genes showed lower but concordant enrichment (Supplementary Figure S2). UMAP visualization further showed that high AUCell signals were mainly distributed in immune cell compartments, whereas relatively low activity was observed in nonimmune populations (Figure 11D). Donor-level statistical analysis further supported the enrichment of this module in macrophages (Figure 11E).

To further characterize hub gene distribution within the myeloid compartment, CD45+ myeloid cells were extracted and reclustered, yielding 12 myeloid subpopulations (Figure 11F). Hub gene module activity was highest in FOLR2^+^/LYVE1+ TRMs and was lower in inflammatory macrophages (Figure 11G–I). These results indicate that the five-gene module is predominantly enriched in cardiac macrophages, particularly in the FOLR2^+^ TRM subset. Therefore, single-cell analysis provided a cell-type context for the five-gene signature identified in bulk myocardium.

### 3.7. Pseudotime and Cell-Communication Analyses Associate the Hub-Gene Module with FOLR2^+^ TRM Cell States

**Figure 12:**
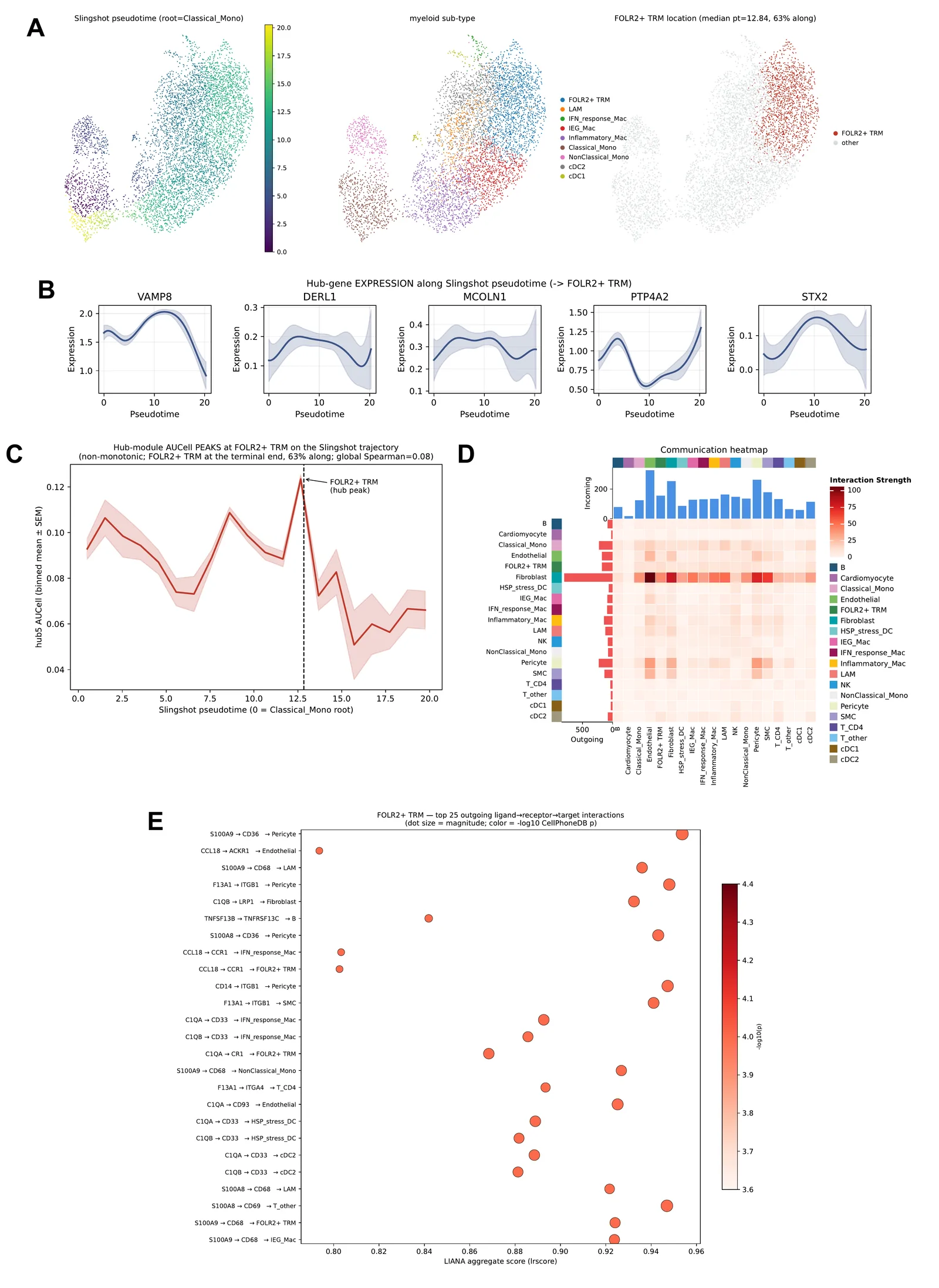
Myeloid pseudotime trajectory and TRM communication analysis. (**A**) Slingshot pseudotime UMAP plot using classical monocytes as the starting population; FOLR2^+^ TRMs are located at the terminal end of the trajectory. (**B**) Expression trends of the five hub genes along pseudotime. (**C**) AUCell activity of the hub gene module along pseudotime, with peak activity observed in FOLR2^+^ TRMs. (**D**) Heatmap showing overall ligand-receptor communication intensity among cardiac cell populations. (**E**) Ligand-receptor interaction analysis between FOLR2^+^ TRMs and representative cardiac cell populations. The shaded regions in panels (**B**,**C**) represent the smoothing applied to the fitted curves. In panel (**D**), color intensity denotes interaction strength; in panel (**E**), dot size denotes interaction magnitude and dot color denotes −log10(*p*).

Given the preferential enrichment of the five-gene module in FOLR2^+^ TRMs, we next investigated its association with myeloid cell-state transitions using pseudotime analysis, with classical monocytes defined as the root population. FOLR2^+^ TRMs were distributed toward the terminal region of the trajectory (Figure 12A). Along pseudotime, the expression levels of the five hub genes and the activity of the corresponding gene module showed an overall upward trend, with higher levels observed in the FOLR2^+^ TRM region (Figure 12B,C). These results indicate that the hub-gene module is associated with FOLR2^+^ TRM cell states along the inferred trajectory. Cell–cell communication analysis further identified potential ligand-receptor interactions between FOLR2^+^ TRMs and fibroblasts, endothelial cells, and other immune cells. These interactions involved complement, chemokine, and matrix-related pathways (Figure 12D,E), indicating that FOLR2^+^ TRMs may participate in immune-stromal crosstalk within the myocardial microenvironment.

To explore potential upstream regulatory mechanisms and drug-related associations, TF-mRNA, miRNA-mRNA, and drug-mRNA networks were constructed using the ChIPBase, TarBase, and CTD databases, respectively. The predicted TFs included SP1, CEBPA, RELA, and STAT3. The predicted miRNAs included hsa-miR-199a-3p, members of the miR-29 family, and members of the miR-15a/16 family. The identified compounds included retinoic acid, cyclosporine, and valproic acid (Supplementary Figure S3). These analyses provide preliminary evidence for further investigation of upstream regulation and potential drug repurposing.

### 3.8. Validation of Hub Gene Downregulation in the TAC-Induced HF Model

To determine whether the hub gene downregulation identified by bulk transcriptomic analysis could be reproduced in vivo, a TAC-induced mouse model of HF was established. Echocardiography showed significant increases in LVEDd, LVEDs, LVEDV, and LVESV in the TAC group compared with the sham group, whereas EF and FS were markedly decreased (Supplementary Figure S4A, Supplementary Table S4). Gross heart size and the LVW/BW ratio were also significantly increased in TAC mice (Supplementary Figure S4, Supplementary Table S4). These findings confirmed that TAC mice developed a typical HF phenotype.

**Figure 13:**
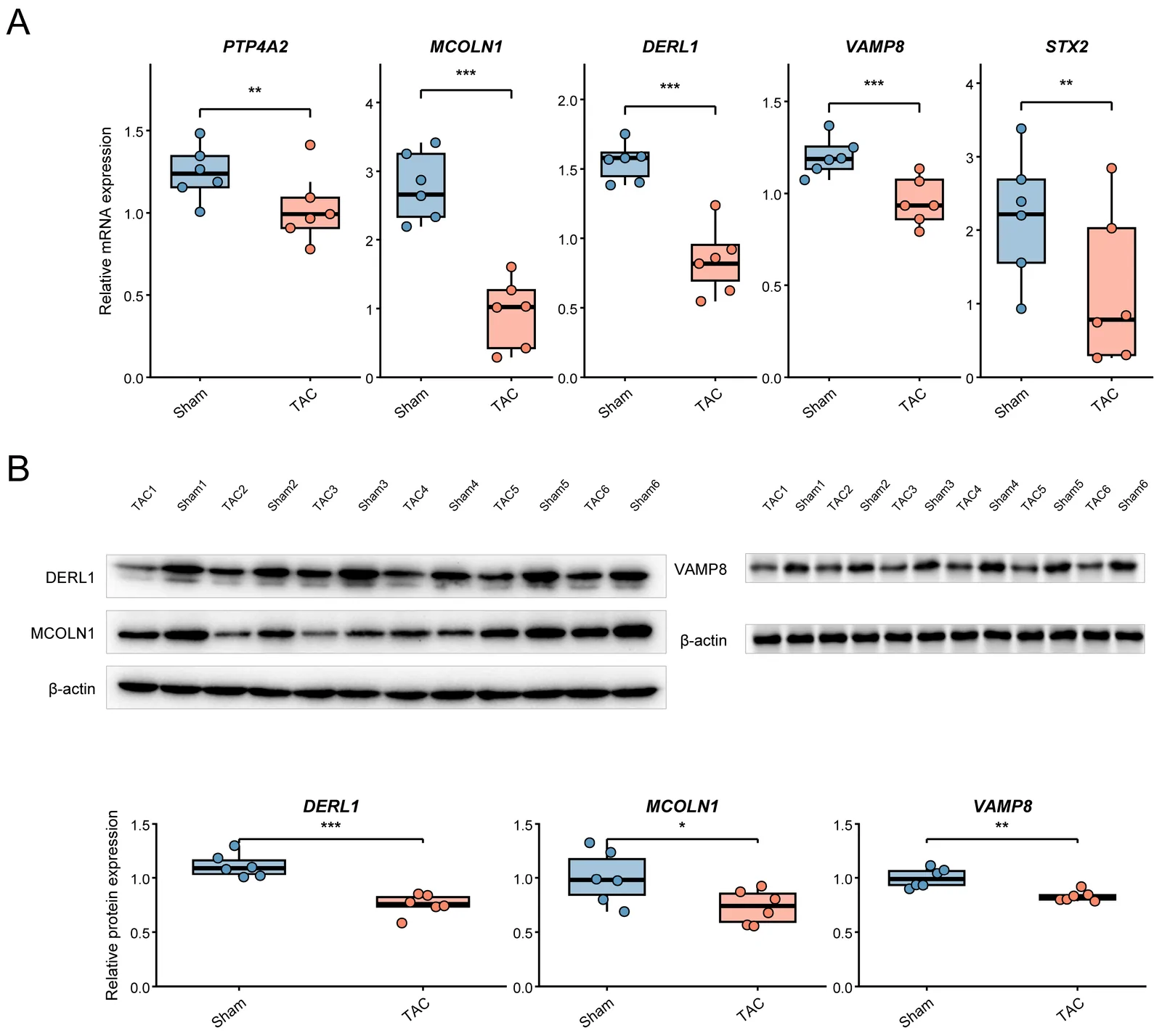
Expression validation of hub genes in the TAC-induced HF mouse model. (**A**) RT-qPCR analysis of hub gene mRNA expression. (**B**) WB analysis and densitometric quantification of protein expression (n = 6 per group). DERL1 and MCOLN1 were analyzed on one blot, whereas VAMP8 was analyzed on a separate blot with its own β-actin loading control. Each target protein was normalized to the corresponding β-actin signal from the same sample and blot. Box-and-whisker plots with individual data points are shown. * *p* < 0.05; ** *p* < 0.01; *** *p* < 0.001.

RT-qPCR analysis showed that the mRNA levels of *PTP4A2*, *MCOLN1*, *DERL1*, *VAMP8*, and *STX2* were significantly lower in TAC myocardium than in sham myocardium (Figure 13A). WB further confirmed reduced *DERL1*, *MCOLN1* and *VAMP8* protein expression in TAC myocardium compared with sham controls, despite inter-animal variability (Figure 13B). These results were consistent with the expression patterns observed in human failing myocardium and support the reproducibility of hub-gene downregulation in pressure-overload-induced HF.

### 3.9. VAMP8 Overexpression Attenuates Ang II/LLOMe-Induced Cardiomyocyte Injury and Lysophagy-Related Protein Alterations

**Figure 14:**
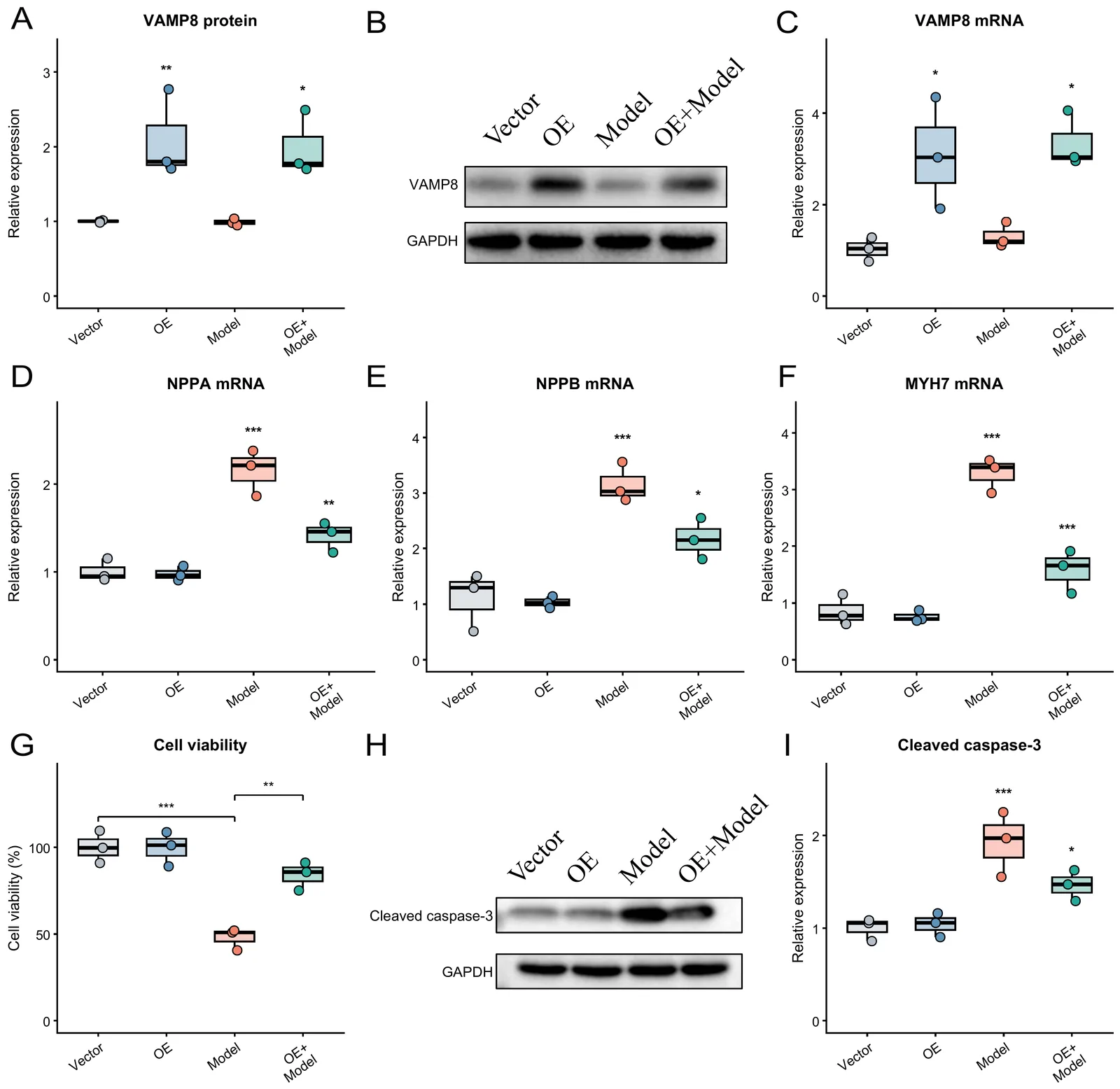
VAMP8 overexpression attenuates Ang II/LLOMe-induced H9c2 cell injury. H9c2 cells were transfected with Vector or OE-VAMP8 plasmids and then treated with Ang II/LLOMe where indicated. (**A**–**C**) Verification of VAMP8 overexpression by Western blot and RT-qPCR. (**D**–**F**) RT-qPCR analysis of *NPPA*, *NPPB*, and *MYH7* expression. (**G**) Cell viability assessed by CCK-8 assay. (**H**,**I**) Western blot analysis of cleaved caspase-3 expression. Box-and-whisker plots display individual data points from three independent biological replicates. * *p* < 0.05; ** *p* < 0.01; *** *p* < 0.001.

With the coordinated downregulation of the five hub genes confirmed in the TAC-induced HF model, we next examined the preliminary functional relevance of a representative candidate. Single-cell analysis indicated that this gene module was predominantly enriched in FOLR2^+^ tissue-resident macrophages, suggesting its potential association with cardiac immune microenvironment homeostasis. Meanwhile, as a SNARE-related molecule, VAMP8 may also participate in cardiomyocyte autophagy–lysosomal homeostasis. VAMP8 was therefore selected from the five hub genes for preliminary functional validation because of its central role in SNARE-mediated fusion and its comparatively strong macrophage-enriched expression. Accordingly, we performed preliminary validation in H9c2 cardiomyocyte-like cells to investigate the effects of VAMP8 on cardiomyocyte injury and lysosome-related protein changes. Results confirmed successful VAMP8 overexpression, with markedly increased VAMP8 levels in the OE-VAMP8 group. VAMP8 expression remained elevated after Ang II/LLOMe stimulation in the OE-VAMP8 + Model group, indicating successful establishment of the VAMP8 overexpression model (Figure 14A–C). Endogenous VAMP8 expression was not significantly altered by Ang II/LLOMe treatment. Thus, the subsequent experiments evaluated whether VAMP8 overexpression could protect H9c2 cells under injury conditions, rather than compensate for a treatment-induced reduction in VAMP8. Ang II/LLOMe treatment significantly upregulated the HF-like markers *NPPA*, *NPPB*, and *MYH7*, reduced cell viability, and increased cleaved caspase-3 expression. These changes were partially reversed by VAMP8 overexpression, suggesting that VAMP8 attenuated Ang II/LLOMe-induced cardiomyocyte injury and apoptosis (Figure 14D–I).

**Figure 15:**
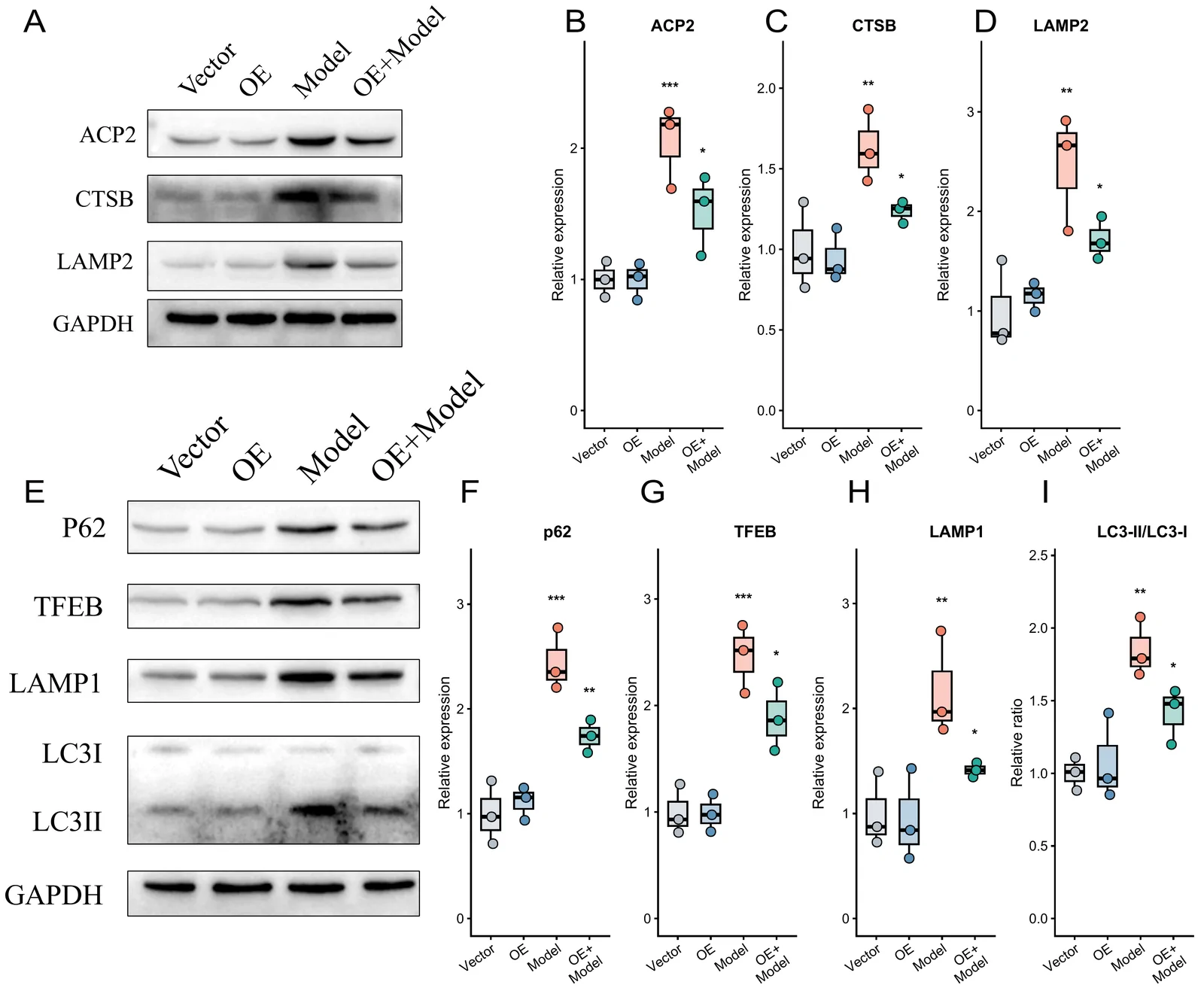
VAMP8 overexpression alleviates Ang II/LLOMe-induced lysosomal and lysophagy-related protein alterations. (**A**–**D**) Western blot analysis and quantification of lysosome-related proteins ACP2, CTSB, and LAMP2. (**E**–**I**) Western blot analysis and quantification of lysophagy-related proteins p62, TFEB, LAMP1, and LC3B-II/I. Box-and-whisker plots display individual data points from three independent biological replicates. * *p* < 0.05; ** *p* < 0.01; *** *p* < 0.001.

To further assess lysosome-related changes, LAMP2, CTSB, and ACP2 expression was examined by WB. Ang II/LLOMe treatment markedly increased the expression of all three proteins, whereas VAMP8 overexpression significantly reduced their expression compared with the Vector + Model group (Figure 15A–D). These results show that VAMP8 overexpression attenuated Ang II/LLOMe-associated changes in the abundance of the measured lysosomal proteins.

Lysophagy-related protein expression was further evaluated by measuring LAMP1, LC3B-II/I, p62, and TFEB. Ang II/LLOMe treatment induced pronounced alterations in these proteins, including increased LAMP1, LC3B-II/I, and TFEB expression and abnormal p62 expression. VAMP8 overexpression attenuated these changes, as shown by reduced LAMP1, LC3B-II/I, and TFEB expression and partial restoration of p62 toward basal levels (Figure 15E–I).

**Figure 16:**
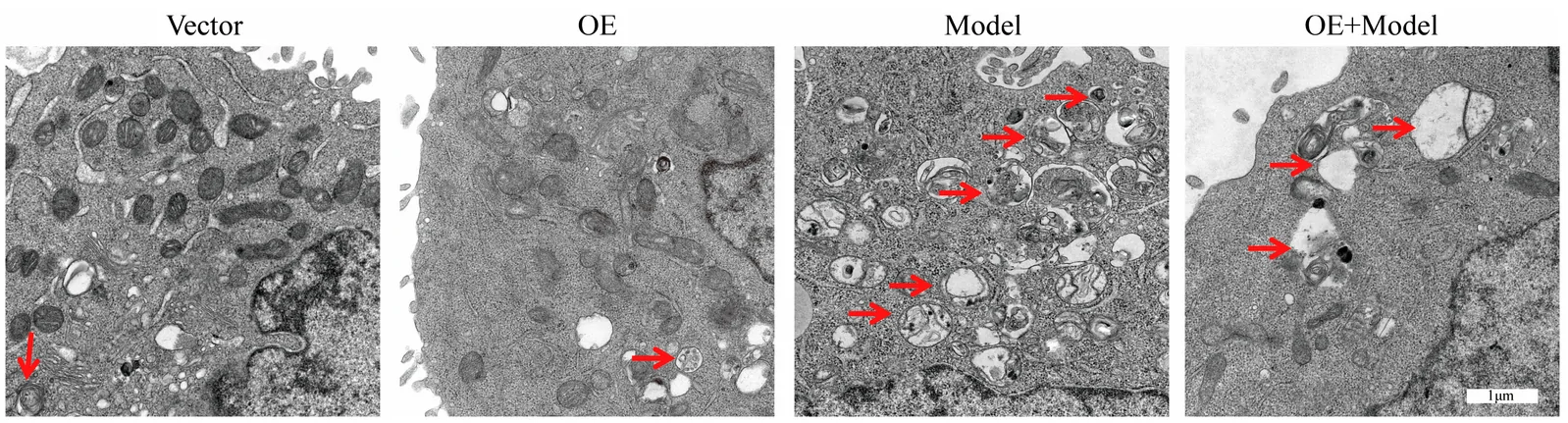
VAMP8 overexpression partially preserves ultrastructural integrity in Ang II/LLOMe-treated H9c2 cells. Representative TEM images showed relatively preserved ultrastructure in Vector and OE cells, marked vacuole-like structure accumulation, lysosome/autolysosome-like structures, mitochondrial swelling, and cytoplasmic damage in Model cells, and partial improvement of these abnormalities in OE + Model cells. Red arrows indicate representative abnormal vacuole-like or lysosome/autolysosome-like structures. TEM images are representative observations and were not included in quantitative statistical analysis. Scale bar = 1.0 μm.

TEM showed preserved ultrastructure in Vector cells, with clearly defined organelles, largely normal mitochondrial morphology, and few lysosome/autolysosome-like or vacuole-like structures (Figure 16). The OE group showed a broadly similar basal ultrastructural appearance, without obvious widespread organelle injury. In contrast, Ang II/LLOMe-treated cells showed marked ultrastructural injury, including increased vacuole-like structures, accumulation of lysosome/autolysosome-like structures, mitochondrial swelling, disrupted cristae, and cytoplasmic organelle damage. Compared with the Model group, the OE + Model group showed fewer abnormal lysosome/autolysosome-like and vacuole-like structures, better-preserved mitochondrial and cytoplasmic integrity. Together, these qualitative observations support partial ultrastructural preservation in the OE + Model group. The OE group showed no obvious widespread basal ultrastructural abnormalities compared with the Vector group.

## 4. Discussion

HF represents a multifactorial clinical condition characterized by inadequate cardiac pumping capacity, which prevents the heart from satisfying the metabolic requirements of peripheral tissues. It continues to impose a substantial public health burden and is strongly associated with mortality and repeated hospital admissions [25]. In this study, myocardial expression profiles from failing hearts were integrated with ML, single-cell transcriptomic analysis, a TAC mouse model, and VAMP8 overexpression experiments to identify lysophagy-related hub genes in HF. Five genes, *VAMP8*, *STX2*, *MCOLN1*, *DERL1*, and *PTP4A2*, were identified as candidate hub genes. Functional enrichment analysis indicated that these genes were mainly associated with SNARE-mediated vesicular transport, autophagosome maturation, and endosomal/lysosomal processes. Single-cell analysis further showed that the candidate gene module was preferentially enriched in cardiac macrophages, particularly FOLR2^+^ TRMs. The *VAMP8* gain-of-function experiment provided preliminary functional support at the cardiomyocyte level. Together, these findings indicate that lysophagy- and vesicular transport-related molecular alterations in failing myocardium may have a distinct cellular basis and may help clarify the contribution of resident macrophages to IME remodeling in HF.

Each analytical layer contributed a distinct part of the overall evidence. Bulk RNA analysis revealed a tissue-level reduction, machine learning narrowed the candidate signature, single-cell analysis identified its predominant cellular distribution, and the TAC model confirmed the corresponding myocardial expression pattern. The VAMP8 experiments then provided preliminary functional evidence for one representative hub gene.

The five hub genes identified in this study are unlikely to act as isolated drivers of HF progression. Rather, they collectively reflect alterations in lysosomal, endosomal, and vesicular transport programs in myocardial tissue, which may represent an important component of pathological cardiac remodeling. Their coordinated decrease may arise from convergent suppression of TFEB-CLEAR-network activity and lysosomal biogenesis, chronic inflammatory or metabolic stress, disrupted SNARE trafficking, changes in resident-macrophage differentiation, or a combination of these processes. VAMP8 is a key SNARE protein involved in lysophagy. It was prioritized for functional validation because its role in autophagosome-lysosome fusion is comparatively direct and its macrophage-enriched single-cell signal was the strongest among the five candidates. VAMP8 contributes to the assembly of the STX17–SNAP29–VAMP8 complex, a key molecular machinery that mediates autophagosome fusion with lysosomes and supports the continuity of autophagic flux [26,27,28]. In addition, post-translational regulation of VAMP8 may affect autophagosome maturation and the stability of myocardial autophagic homeostasis [29,30]. In this study, cardiomyocyte injury was modeled in vitro through combined Ang II and LLOMe stimulation to investigate whether VAMP8 protects against lysosomal impairment and lysophagy-associated cellular damage. The findings indicate that VAMP8 overexpression was associated with reduced cardiomyocyte injury, attenuation of lysosome- and autophagy-related protein alterations, and fewer ultrastructural abnormalities under injury conditions.

STX2 is a major syntaxin/SNARE family member and plays an essential role in intracellular vesicle trafficking and membrane fusion [31]. Consistent with the enrichment of STX2-related pathways in vesicular transport and energy metabolism, STX2 downregulation may reflect impaired organelle homeostasis and altered metabolic adaptation in failing cardiomyocytes. However, whether STX2 directly regulates myocardial energy metabolism requires further validation [32]. MCOLN1 is a lysosomal calcium channel that contributes to lysosomal calcium homeostasis and acidification balance [33]. DERL1 participates in endoplasmic reticulum-associated degradation and protein quality control, indicating cellular stress and proteostasis abnormalities in HF [34]. PTP4A2 has been reported to promote lysophagy by dephosphorylating VCP/p97 at Tyr805 and facilitating its interaction with cofactors such as UBXN6/UBXD1 and PLAA, thereby contributing to damaged lysosome clearance and lysosomal homeostasis [35]. Its inclusion in the present hub gene set suggests that PTP4A2 may also participate in lysosomal/vesicular transport and broader cellular homeostatic programs. In addition, the diagnostic model based on these five hub genes showed favorable discriminative performance in both the training set and the small external validation set, supporting their potential biomarker value. Nevertheless, because the validation cohort was limited in size, the diagnostic stability and clinical translational value of this model should be further evaluated in larger, independent, prospective clinical cohorts.

Pathway enrichment and immune infiltration analyses indicated marked stromal remodeling and immune microenvironmental alterations in failing myocardium. GSEA showed significant changes in WNT signaling, TGF-β/EMT, and inflammatory/interferon response gene sets, suggesting that HF may be accompanied by extracellular matrix remodeling, fibroblast activation, and dysregulated inflammatory and immune programs. Previous studies have shown that extracellular matrix remodeling and cardiac fibrosis are central pathological features of both ischemic and nonischemic HF progression, and that TGF-β and WNT signaling are key pathways involved in cardiac fibrosis and remodeling [36,37,38,39]. Immune infiltration analysis further showed altered relative abundances of multiple immune cell types in HF samples. Hub gene expression was correlated with M2 macrophages, activated CD8+ T cells, and other immune subpopulations [40,41], indicating that the hub gene module is embedded within the altered IME of HF. However, immune deconvolution based on bulk transcriptomic data can only estimate relative changes in immune cell composition. It cannot establish direct regulation of immune cell infiltration (ICI), polarization, or activation by the candidate genes. Further single-cell and functional experiments are therefore needed to define these relationships.

Single-cell analysis provided further evidence regarding the cellular sources of the candidate gene module. Cardiac resident macrophages include embryonically derived CCR2− populations that can self-renew locally and contribute to tissue homeostasis, whereas recruited CCR2+ monocyte-derived macrophages more commonly expand during injury and inflammatory remodeling [42]. Among cardiac cell populations, the module was preferentially enriched in macrophages, most prominently in myeloid FOLR2^+^ TRMs, and showed lower activity in inflammatory macrophages. Previous studies have shown that TRMs have relatively conserved developmental origins and turnover dynamics across organs [43]. FOLR2/FRβ is also an important marker of TRMs and macrophages in inflamed tissues [44]. Pseudotime analysis further showed that hub gene module activity varied along the inferred trajectory rooted in classical monocytes, with higher activity in the FOLR2^+^ TRM region. Cell–cell communication analysis suggested that FOLR2^+^ TRMs may interact with nonimmune cells, including fibroblasts, endothelial cells, and smooth muscle cells, through complement, chemokine, and matrix-related signaling pathways. Together with previous evidence that FOLR2^+^ macrophages can participate in tissue fibrosis and matrix remodeling [45], these findings suggest that high hub gene module activity may represent a characteristic molecular feature of cardiac FOLR2^+^ TRMs.

Coordinated downregulation of these hub genes in HF may reflect intrinsic suppression of the lysosomal/vesicular program within resident macrophages, depletion or phenotypic conversion of FOLR2^+^ TRMs, replacement by recruited monocyte-derived macrophages, or a combination of these processes. It should be noted that the single-cell and in vitro experiments in this study address different biological levels. The H9c2 gain-of-function experiment does not directly demonstrate that VAMP8 regulates FOLR2^+^ TRM function, but it provides preliminary evidence for a potential cardiomyocyte-level effect. Spatial transcriptomics, multiplex immunofluorescence, or RNAscope will be needed to confirm the anatomical distribution of FOLR2^+^ macrophages and individual hub genes in failing myocardium.

Exploratory network analyses suggested several potential upstream regulators and drug-related associations of the five hub genes. Some of the predicted miRNAs and compounds have previously been implicated in autophagy, fibrosis, cardiac remodeling, and cardiovascular pathology [46,47,48,49,50]. However, these findings were derived from database-based predictions and should be regarded as hypotheses for future mechanistic validation.

In addition, hub gene expression changes were validated in a TAC-induced mouse model of HF. The mRNA levels of all five hub genes were significantly lower in TAC myocardium than in sham myocardium, and DERL1, MCOLN1 and VAMP8 protein levels were also decreased, supporting the overall downregulation trend.

This study has several limitations. First, the analyses were retrospective and based on public databases. Although batch correction was performed, heterogeneity in sample collection time, processing protocols, and baseline patient characteristics may have introduced bias. Second, the external validation set was small, and the diagnostic stability of the hub gene model requires further evaluation in larger multicenter clinical cohorts. Third, the number of normal control samples in the single-cell dataset was limited; therefore, comparisons across disease states should be interpreted with caution. In addition, pseudotime and cell–cell communication analyses were exploratory and require further experimental validation. Fourth, although the TAC mouse model recapitulates pressure overload-induced HF, it does not fully reflect the complex pathophysiology of human HF. Fifth, the VAMP8 overexpression experiments did not include Ang II-only or LLOMe-only treatment groups, preventing separation of the independent effects of Ang II and LLOMe on lysophagy-related changes. Although VAMP8 was downregulated in human HF myocardial tissues and TAC mouse hearts, Ang II/LLOMe-treated H9c2 cells did not fully reproduce this endogenous downregulation, suggesting that VAMP8 reduction in vivo may be related to chronic pressure overload, multicellular myocardial microenvironment, or cell type-specific regulation. Therefore, the H9c2 overexpression experiment was designed as a preliminary functional validation rather than a complete recapitulation of the in vivo expression pattern. In addition, although VAMP8 overexpression in H9c2 cells provided preliminary evidence for its cardiomyocyte-protective role, this experiment did not directly validate the function of VAMP8 in FOLR2^+^ TRMs. Future studies using macrophage-specific models, coculture systems, or spatial validation approaches are required to clarify whether VAMP8 regulates FOLR2^+^ TRM-mediated immune–stromal interactions in HF.

The addition of an OE-VAMP8-only group allowed for the assessment of the basal ultrastructural effect of VAMP8 overexpression. OE cells showed a broadly similar ultrastructural appearance to Vector cells, without obvious widespread organelle injury. This comparison supports the interpretation that the differences between the Model and OE + Model groups were observed mainly under injury conditions. Nevertheless, the TEM findings remain qualitative supportive evidence and do not establish a definitive rescue effect. In addition, changes in the abundance of lysosome- or autolysosome-like structures cannot by themselves establish altered lysophagy flux, which will require direct flux assays and lysosome-damage-specific readouts. Similarly, static changes in LC3B-II/I and p62/SQSTM1 expression do not directly indicate restoration of lysophagy flux.

Finally, this study mainly validated expression changes in the hub genes and did not directly determine their causal roles in FOLR2^+^ TRM function, autophagic flux, vesicular transport, or cardiac remodeling. Future studies should include large-scale clinical cohorts, spatial localization analyses, FOLR2^+^ TRM-specific functional experiments, Galectin-3 puncta detection, LAMP1/LC3B colocalization analysis, and knockdown, overexpression, or rescue experiments targeting the remaining candidate genes to further validate the mechanistic hypotheses proposed in this study.

## 5. Conclusions

This study identified five HF-related hub genes, *VAMP8*, *STX2*, *MCOLN1*, *DERL1*, and *PTP4A2*, all of which were consistently downregulated in failing myocardium and closely associated with SNARE-mediated vesicular transport, autophagosome maturation, and endosomal/lysosomal processes. A diagnostic model based on these genes showed favorable discriminative performance and potential biomarker value. Single-cell analysis localized this gene module mainly to cardiac FOLR2^+^ TRMs, suggesting alterations in resident macrophage-associated lysosomal and vesicular transport programs in failing myocardium. The TAC mouse model further validated the downregulation of these hub genes in failing myocardium. The *VAMP8* overexpression results suggested that *VAMP8* may reduce cardiomyocyte injury, with attenuated changes in lysosomal- and lysophagy-related protein abundance and partial preservation of cellular ultrastructural integrity. Overall, this study identifies candidate molecular markers for HF and associates a lysophagy- and vesicular-transport-related gene module with FOLR2^+^ TRMs and predicted immune–stromal communication in failing myocardium.

## Figures and Tables

**Table 1:** Basic Information of the Included GEO Datasets.

Dataset	GSE16499	GSE57338	GSE76701	GSE145154
Platform	GPL5175	GPL11532	GPL570	GPL20795/GPL24676
Species	Human	Human	Human	Human
Tissue	Left ventricle	Left ventricle	Left ventricle	Left ventricle
HF samples	15	177	4	5
Control samples	15	136	4	1
Reference (PMID)	20,124,440	25,528,681	26,756,417	34,601,654

**Table 2:** RT-qPCR Primer Sequences.

Gene Symbol	Forward Primer (5′ → 3′)	Reverse Primer (5′ → 3′)
Mouse *PTP4A2*	TGATTGAATGCGGAATGAAG	TGGTATCTCTGAAGCGTAAC
Mouse *MCOLN1*	GGACTTGGACTTCTTGGAT	CATTGTTGATGAGGCTCTG
Mouse *DERL1*	GGACTGGTTCAGGAGCAT	TGGAAGCGATAGAGGAAGG
Mouse *VAMP8*	AAGACAGAGGACTTGGAAG	GAGGATGACGATGATAAGGA
Mouse *STX2*	TCATCCTGTCTGCTCCAA	CGCTTCATTGTATTCTGTCA
Rat *NPPA*	CGTGCCCCGACCCACGCC	GGCTCCGAGGGCCAGCGAG
Rat *NPPB*	AGAACAATCCACGATGCAGAAG	AAACAACCTCAGCCCGTCACA
Rat *MYH7*	GAGCCTCCAGAGTTTGCTGAAGGA	CTGCAGTCGCAGTAGGTTCT
Rat *VAMP8*	CACTTCAAGACGACGTCCCA	GGTGCCCGTAGCAAAGAGTA

## Data Availability

The public datasets analyzed in this study are available in the NCBI Gene Expression Omnibus (GEO) database. The bulk myocardial transcriptome datasets are available under accession numbers GSE16499, GSE57338, and GSE76701, and the human cardiac single-cell RNA-seq dataset is available under accession number GSE145154.

## Supplementary Materials

The following supporting information can be downloaded at https://www.mdpi.com/article/10.3390/genes17080957/s1, Figure S1: Single-gene GSEA of hub genes. A–E, GSEA enrichment curves for *VAMP8* (A), *DERL1* (B), *MCOLN1* (C), *PTP4A2* (D), and *STX2* (E); Figure S2: Individual hub-gene expression in the cardiac single-cell atlas. A, UMAP feature plots for *VAMP8*, *DERL1*, *MCOLN1*, *PTP4A2*, and *STX2*, together with the five-gene AUCell module. B, Dot plot showing mean expression and detection fraction of the five hub genes across major cardiac cell types; Figure S3: Regulatory networks and drug associations of hub genes. A, mRNA–TF regulatory network based on ChIPBase. B, mRNA–miRNA regulatory network based on TarBase. C, mRNA–drug association network based on CTD; Figure S4: Validation of the TAC-induced mouse HF model. A, M-mode echocardiography and quantitative analysis of cardiac function parameters, including EF, FS, LVIDd, and LVIDs, in the sham and TAC groups. B, Gross heart morphology and LVW/BW ratio; Table S1: List of lysophagy-related genes used in this study. The complete table is provided as a separate CSV file; Table S2: Detailed GO and KEGG results of LRDEGs; Table S3: Detailed GSEA results in the integrated dataset; Table S4: Cardiac parameters in each group.
