# Supplementary material for: Integrated Bulk and Single-Cell Transcriptomic Analyses Identify a FOLR2+ Tissue-Resident Macrophage-Associated Lysophagy Gene Module in Heart Failure
Source: Genes (Basel). 2026 Aug 15;17(8):957. doi: 10.3390/genes17080957 (PMC13512565; doi:10.3390/genes17080957)
Supplement: Supplementary file 1 [file genes-17-00957-s001.zip › Supplementary_Figures_and_Tables.pdf]

Supplementary Figures and Tables

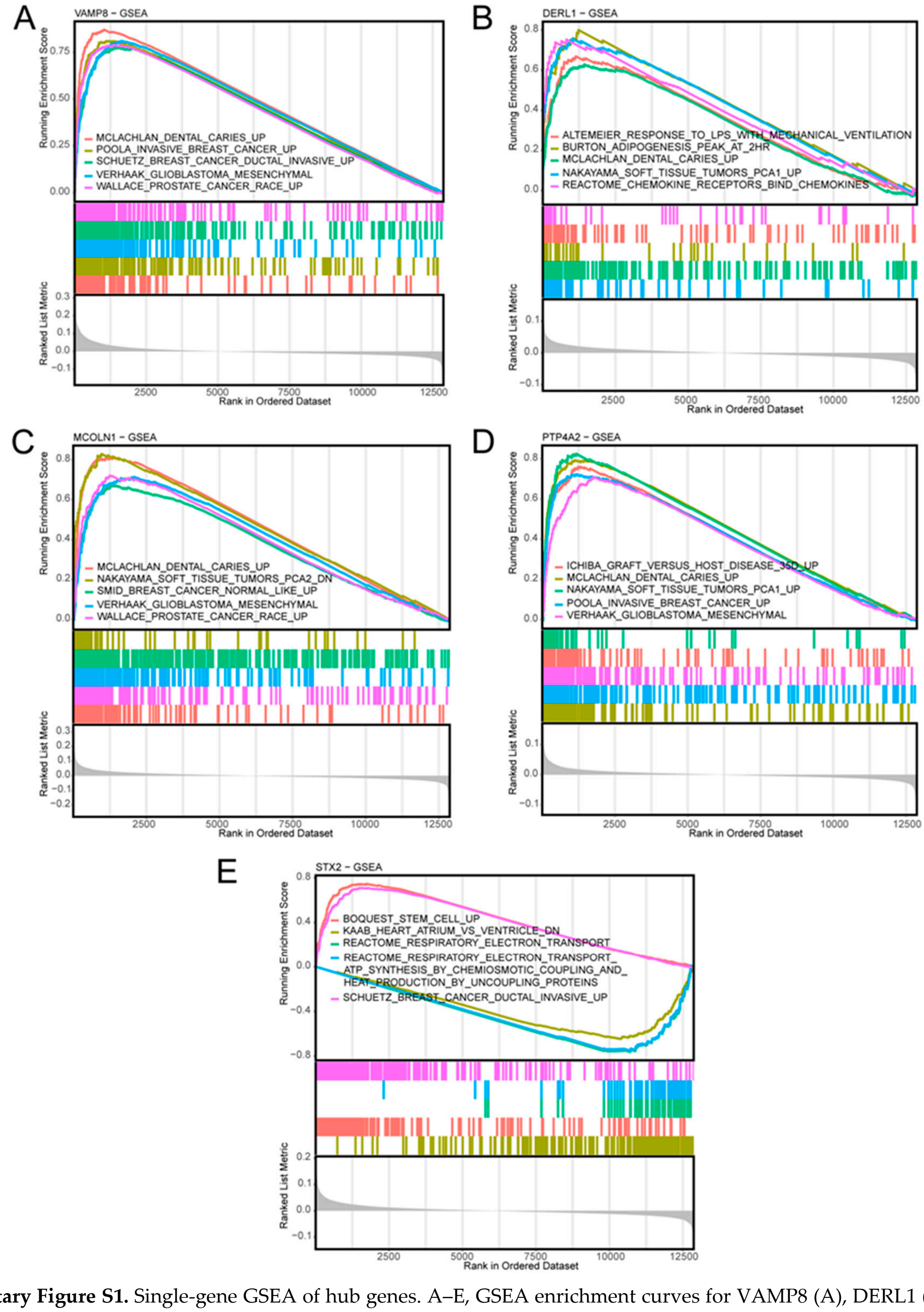

Supplementary Figure S1. Single-gene GSEA of hub genes. A–E, GSEA enrichment curves for VAMP8 (A), DERL1 (B), MCOLN1 (C), PTP4A2 (D), and STX2 (E).

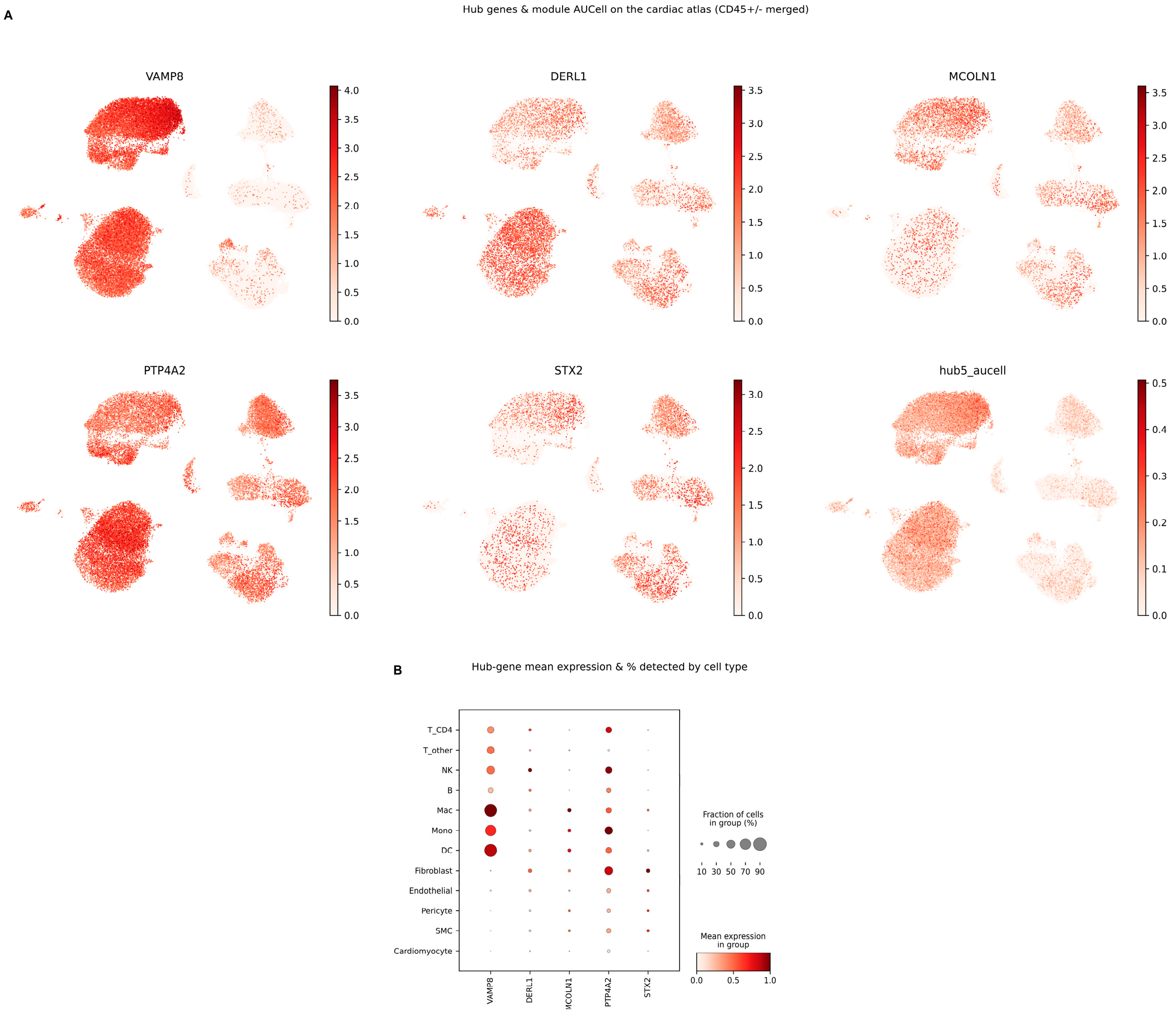

Supplementary Figure S2. Individual hub-gene expression in the cardiac single-cell atlas. A, UMAP feature plots for VAMP8, DERL1, MCOLN1, PTP4A2, and STX2, together with the five-gene AUCell module. B, Dot plot showing mean expression and detection fraction of the five hub genes across major cardiac cell types.

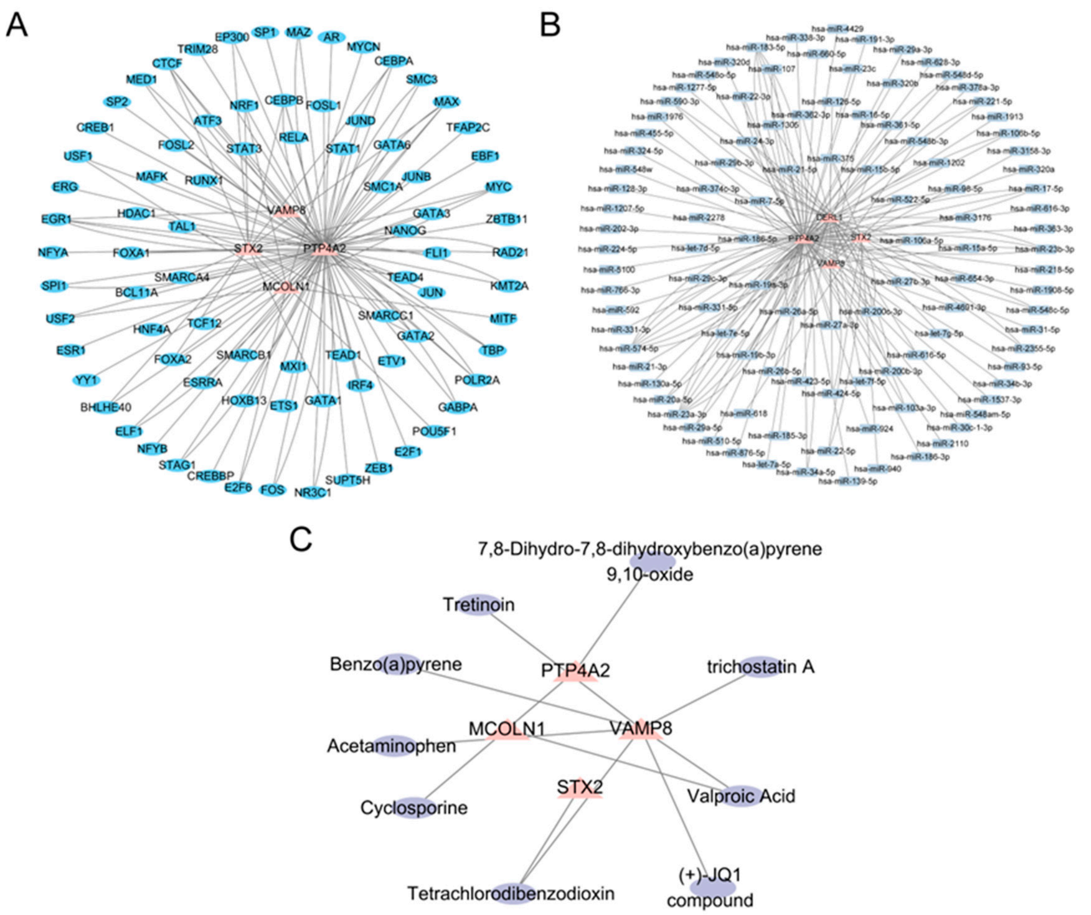

Supplementary Figure S3. Regulatory networks and drug associations of hub genes. A, mRNA-TF regulatory network based on ChIPBase. B, mRNA-miRNA regulatory network based on TarBase. C, mRNA-drug association network based on CTD.

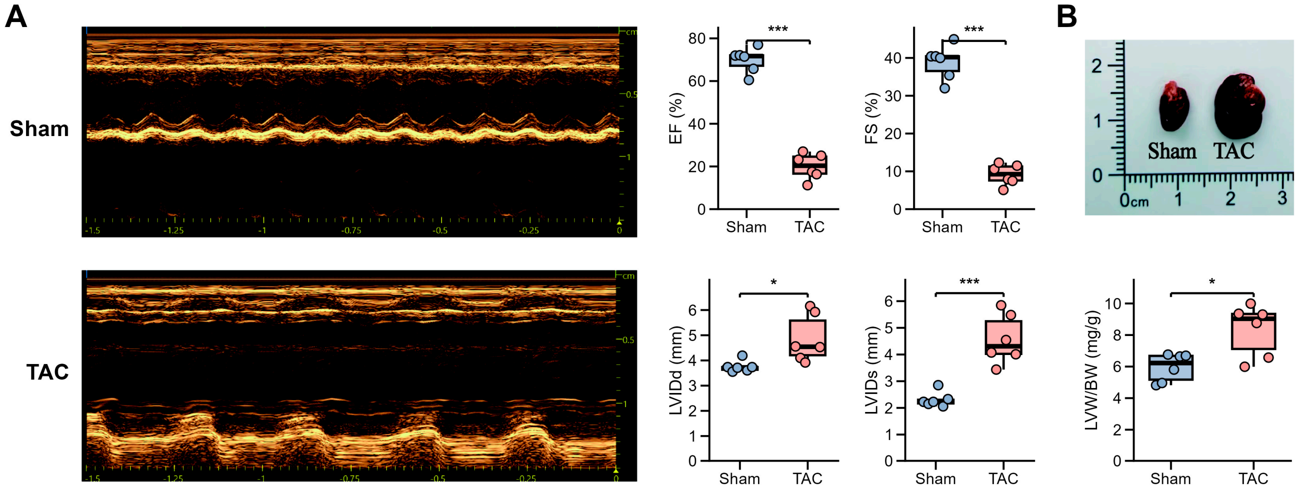

Supplementary Figure S4. Validation of the TAC-induced mouse HF model. A, M-mode echocardiography and quantitative analysis of cardiac function parameters, including EF, FS, LVIDd, and LVIDs, in the sham and TAC groups. B, Gross heart morphology and LVW/BW ratio.

Supplementary Table S1. List of lysophagy-related genes used in this study. The complete table is provided as a separate CSV file.

Supplementary Table S2. Detailed GO and KEGG Results of LRDEGs.

	Category	Term Description	*P* value	*q* value
GO:0002886	BP	regulation of myeloid leukocyte mediated immunity	1.430E-04	5.926E-03
GO:0097352	BP	autophagosome maturation	1.430E-04	5.926E-03
GO:0140029	BP	exocytic process	2.767E-04	5.926E-03
GO:0140056	BP	organelle localization by membrane tethering	2.767E-04	5.926E-03
GO:0022411	BP	cellular component disassembly	3.423E-04	5.926E-03
GO:0005770	CC	late endosome	7.600E-05	9.400E-04
GO:0031201	CC	SNARE complex	8.505E-05	9.400E-04
GO:0005769	CC	early endosome	1.874E-04	1.311E-03
GO:0030670	CC	phagocytic vesicle membrane	2.372E-04	1.311E-03
GO:0045335	CC	phagocytic vesicle	7.453E-04	2.801E-03
GO:0005484	MF	SNAP receptor activity	5.811E-05	1.040E-03
GO:0000149	MF	SNARE binding	4.899E-04	4.383E-03
GO:0017081	MF	chloride channel regulator activity	4.210E-03	1.632E-02
GO:0015278	MF	calcium-release channel activity	5.826E-03	1.632E-02
GO:0042288	MF	MHC class I protein binding	6.471E-03	1.632E-02
hsa04130	KEGG	SNARE interactions in vesicular transport	1.305E-04	2.748E-03

Supplementary Table S3. Detailed GSEA Results in the Integrated Dataset.

Gene Set Name	NES	adjusted *P*	*q* value
KEGG_MEDICUS_REFERENCE_WNT_SIGNALING_MODULATION_WNT_INHIBITOR	2.211E+00	3.220E-05	2.690E-05
KRASNOSELSKAYA_ILF3_TARGETS_UP	2.136E+00	1.126E-04	9.430E-05
FOROUTAN_INTEGRATED_TGFB_EMT_DN	-1.816E+00	5.775E-03	4.834E-03
SCHOEN_NFKB_SIGNALING	-1.852E+00	1.271E-02	1.064E-02
IZADPANAH_STEM_CELL_ADIPOSE_VS_BONE_DN	2.293E+00	2.060E-07	1.725E-07
ANASTASSIOU_MULTICANCER_INVASIVENESS_SIGNATURE	2.273E+00	1.653E-06	1.383E-06
TURASHVILI_BREAST_LOBULAR_CARCINOMA_VS_DUCTAL_NORMAL_UP	2.246E+00	4.175E-06	3.494E-06
TAKEDA_TARGETS_OF_NUP98_HOXA9_FUSION_8D_UP	2.245E+00	7.951E-08	6.656E-08
RICKMAN_HEAD_AND_NECK_CANCER_B	2.231E+00	1.198E-05	1.003E-05
MOSERLE_IFNA_RESPONSE	2.224E+00	6.950E-06	5.818E-06
NABA_PROTEOGLYCANS	2.220E+00	1.903E-05	1.593E-05
NABA_CORE_MATRISOME	2.153E+00	7.951E-08	6.656E-08
MOLENAAR_TARGETS_OF_CCND1_AND_CDK4_UP	2.136E+00	1.304E-04	1.091E-04
TAKEDA_TARGETS_OF_NUP98_HOXA9_FUSION_3D_UP	2.120E+00	1.175E-06	9.833E-07
BROWNE_INTERFERON_RESPONSIVE_GENES	2.111E+00	9.880E-05	8.270E-05
SEITZ_NEOPLASTIC_TRANSFORMATION_BY_8P_DELETION_UP	2.106E+00	8.516E-05	7.128E-05

Gene Set Name	NES	adjusted *P*	*q* value
WAMUNYOKOLI_OVARIAN_CANCER_LMP_DN	2.106E+00	8.701E-07	7.283E-07
PLASARI_NFIC_TARGETS_BASAL_UP	2.100E+00	2.946E-04	2.466E-04

Supplementary Table S4. Cardiac Parameters in Each Group.

Parameter	Sham (n=6)	TAC (n=6)	Statistical difference
BW/g	22.35 ± 0.41	22.93 ± 0.77	n.s.
LVW/BW/(mg·g^{-1})	5.95 ± 0.89	8.33 ± 1.64	$P < 0.05$
LVEDd/mm	3.76 ± 0.22	5.00 ± 0.87	$P < 0.01$
LVEDs/mm	2.31 ± 0.28	4.56 ± 0.93	$P < 0.01$
EF/%	69.79 ± 5.80	20.04 ± 6.01	$P < 0.01$
FS/%	38.86 ± 4.53	9.14 ± 2.77	$P < 0.01$
LVW/mg	132.97 ± 20.62	191.27 ± 40.20	$P < 0.05$
LVEDV/μl	60.83 ± 8.79	122.66 ± 49.32	$P < 0.05$
LVESV/μl	18.65 ± 6.23	100.40 ± 47.39	$P < 0.05$
